# Next-generation phenotyping of inherited retinal diseases from multimodal imaging with Eye2Gene

**DOI:** 10.1038/s42256-025-01040-8

**Published:** 2025-06-18

**Authors:** Nikolas Pontikos, William A. Woof, Siying Lin, Biraja Ghoshal, Bernardo S. Mendes, Advaith Veturi, Quang Nguyen, Behnam Javanmardi, Michalis Georgiou, Alexander Hustinx, Miguel A. Ibarra-Arellano, Ismail Moghul, Yichen Liu, Kristina Pfau, Maximilian Pfau, Mital Shah, Jing Yu, Saoud Al-Khuzaei, Siegfried K. Wagner, Malena Daich Varela, Thales Antonio Cabral de Guimarães, Sagnik Sen, Gunjan Naik, Dayyanah Sumodhee, Dun Jack Fu, Nathaniel Kabiri, Jennifer Furman, Bart Liefers, Aaron Y. Lee, Samantha R. De Silva, Caio Marques, Fabiana Motta, Yu Fujinami-Yokokawa, Alison J. Hardcastle, Gavin Arno, Birgit Lorenz, Philipp Herrmann, Kaoru Fujinami, Juliana Sallum, Savita Madhusudhan, Susan M. Downes, Frank G. Holz, Konstantinos Balaskas, Andrew R. Webster, Omar A. Mahroo, Peter M. Krawitz, Michel Michaelides

**Affiliations:** 1 University College London Institute of Ophthalmology, University College London, London, UK; 2 Moorfields Eye Hospital, London, UK; 3 Institute for Genomic Statistic and Bioinformatics, University Hospital Bonn, Rheinische-Friedrich-Wilhelms University, Bonn, Germany; 4 Department of Ophthalmology, University Hospital Bonn, Rheinische-Friedrich-Wilhelms Universität Bonn, Bonn, Germany; 5 Department of Ophthalmology, University Hospital Basel, Basel, Switzerland; 6 Oxford Eye Hospital, John Radcliffe Hospital, Oxford, UK; 7 Nuffield Department of Clinical Neurosciences, John Radcliffe Hospital, Oxford, UK; 8 Department of Ophthalmology, Faculdade São Leopoldo Mandic, Campinas, São Paulo, Brazil; 9 UCL Translational Research Office, UCL Maple House, London, UK; 10 Department of Ophthalmology, University of Washington School of Medicine, Seattle, WA USA; 11 Roger and Angie Karalis Johnson Retina Center, University of Washington, Seattle, WA USA; 12 Department of Ophthalmology and Visual Sciences, Escola Paulista de Medicina, Federal University of Sao Paulo, São Paulo, Brazil; 13 Laboratory of Visual Physiology, Division of Vision Research, National Institute of Sensory Organs, National Hospital Organization Tokyo Medical Center, Tokyo, Japan; 14 St Paul’s Eye Unit, The Royal Liverpool and Broadgreen University Hospitals, Liverpool, UK

**Keywords:** Image processing, Molecular medicine, Genetic testing, Retinal diseases

## Abstract

Rare eye diseases such as inherited retinal diseases (IRDs) are challenging to diagnose genetically. IRDs are typically monogenic disorders and represent a leading cause of blindness in children and working-age adults worldwide. A growing number are now being targeted in clinical trials, with approved treatments increasingly available. However, access requires a genetic diagnosis to be established sufficiently early. Critically, the timely identification of a genetic cause remains challenging. We demonstrate that a deep learning algorithm, Eye2Gene, trained on a large multimodal imaging dataset of individuals with IRDs (*n* = 2,451) and externally validated on data provided by five different clinical centres, provides better-than-expert-level top-five accuracy of 83.9% for supporting genetic diagnosis for the 63 most common genetic causes. We demonstrate that Eye2Gene’s next-generation phenotyping can increase diagnostic yield by improving screening for IRDs, phenotype-driven variant prioritization and automatic similarity matching in phenotypic space to identify new genes. Eye2Gene is accessible online (app.eye2gene.com) for research purposes.

## Abstract (web summary)

Eye2Gene’s next-generation phenotyping of multimodal images increases diagnostic yield for inherited retinal diseases by improving screening, phenotype-driven variant prioritization and automatic similarity matching in phenotypic space to drive gene discovery.

## Main

Inherited retinal diseases (IRDs) are a group of rare monogenic conditions affecting 1 in 3,000 people, with more than 270 different IRD-associated genes identified so far^1–3,4^. IRDs cause degeneration of the retina, the light-sensitive tissue at the back of the eye responsible for vision. Some individuals with IRDs may be profoundly visually impaired from birth, while others experience progressive peripheral and/or central vision deterioration over time. Cumulatively, IRDs are a leading cause of blindness in children and the working-age population, with a substantial psychological and socioeconomic impact^5^.

Revealing the genetic cause of an IRD is a prerequisite to optimally determining prognosis, providing genetic counselling and inclusion in gene-directed clinical trials. However, this genetic diagnosis remains elusive in more than 40% of cases on average according to studies conducted in the UK^6–8^, and the rate of diagnosis is likely to be much lower in parts of the world where genetic testing is less widely available^9,10^. This is mostly due to limited access, lack of resources and infrastructure to support genetic testing services, inefficiencies in their provision and a shortage of specialists who can interpret the findings from molecular tests^11^.

IRDs often have distinct phenotypic features that clinicians learn to recognize aided by modern high-resolution retinal imaging technology that rapidly and non-invasively acquires images of the retina with minimal inconvenience to the patient. These scans can be performed by a variety of imaging modalities such as fundus autofluorescence (FAF), infrared (IR) reflectance imaging and spectral-domain optical coherence tomography (SD-OCT), each of which convey different information about retinal health and architecture (Supplementary Fig. 1). FAF images can yield data relating to outer retinal and retinal pigment epithelium (RPE) health. Hyperautofluorescence in FAF images can result from either accumulation of fluorescent material (including lipofuscin) or from loss of photoreceptor outer segments or macular luteal pigment (which usually absorb the incoming short wavelengths), while loss of autofluorescence can be associated with loss of the RPE. These patterns are associated with particular IRDs^12^. IR images are usually acquired together with SD-OCT scans. Brightness in IR images can be associated with levels of melanin and some early lesions associated with certain IRDs, such as pattern dystrophies, can be more apparent on IR than on FAF^13^. SD-OCT gives a high-resolution cross-sectional image of the retinal layers (including photoreceptor outer segments, external limiting membrane, outer and inner plexiform and nuclear layers, ganglion cell and nerve fibre layers) and the RPE. Photoreceptors are the primary cell type affected in many IRDs, and the reflectivity and width of the hyperreflective ellipsoid zone (the mitochondria-rich portion of the inner segment of the photoreceptors) is used to assess photoreceptor integrity and as a marker for disease progression^14^.

This high-resolution in-depth multimodal imaging information enables ophthalmologists to identify gene-specific patterns of disease, allowing prediction of the disease-associated gene in some cases. However, given the sparsity of these diseases individually, the experience required to make accurate clinical diagnoses is not widely available and limited to a handful of specialists and clinics who have developed this expertise over several decades. Wider access to this expertise could be deployed via an AI system trained to detect gene-specific patterns from multimodal retinal imaging scans.

Due to transformational improvements in imaging technology and a comprehensive genetic testing framework for IRDs embedded in specialist healthcare services over the past decade in the UK^15^, there are now a sufficient number of genetically characterized patients with detailed retinal phenotyping to build representative datasets for deep learning. Moorfields Eye Hospital (MEH) in the UK currently provides one of the most extensive datasets in the world^1^.

We have leveraged this resource to develop a deep learning model, Eye2Gene, able to predict the causative IRD gene from the retinal scans of a patient with an IRD, acquired using the three aforementioned imaging modalities of FAF, IR and SD-OCT. Eye2Gene was trained on retinal scans acquired in individuals with IRDs seen at MEH who had undergone genetic testing and where a confirmed genetic cause had been identified by an accredited diagnostic laboratory. Eye2Gene was internally validated on a held-out set of retinal scans from MEH, and externally validated on retinal scans from individuals with IRDs from five different external clinical centres. Eye2Gene performance was also compared to that of expert clinicians.

## Results

**Fig. 1:**
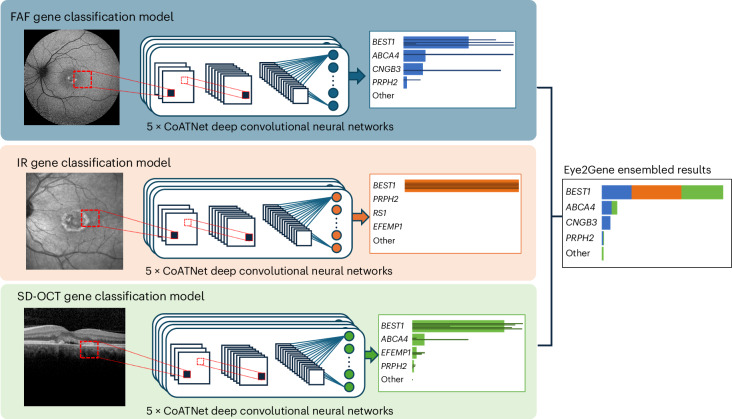
Eye2Gene model. Eye2Gene provides IRD-gene prediction given a retinal scan of one of three imaging modalities (FAF, IR or SD-OCT) for up to 63 gene classes. Images are initially resized to 256 by 256 pixels and rescaled to the range [0,1]. Each image modality-specific predictor block consists of an ensemble of five CoAtNet neural networks. The outputs are averaged to produce a final prediction output. The performance of Eye2Gene is evaluated on a held-out internal test dataset from MEH consisting of 28,174 images acquired from 524 patients over 9,291 patient visits since 2006.

Eye2Gene is an ensemble of a total of 15 constituent CoAtNet deep convolutional neural networks, which takes one or more retinal scans of three different imaging modalities from a given patient and outputs a gene-level prediction score for 63 distinct IRD genes^16^. Collectively, these 63 genes cover over 90% of genetically characterized IRD cases in the European population^1,4,17–19^. Given approximately 60–70% of IRD cases are molecularly diagnosed followed genetic testing, this may represent 54–63% of the total IRD population that includes both diagnosed and undiagnosed patients^10^. The Eye2Gene model was trained on a total of 58,030 scans from 2,451 patients (4,801 eyes, 9,291 appointments) from MEH, split into three different modalities: FAF (*n* = 16,708), IR (*n* = 20,659) and SD-OCT volumes (*n* = 20,663). For each of the three modalities, five distinct CoAtNet deep convolutional neural networks were trained, resulting in a total of 15 neural networks with identical architecture but different network weights (Supplementary Fig. 2). For each modality, these five networks were then combined into three modality-specific models by ensembling. The combination of these three models constitutes Eye2Gene. Given a single input scan of one of the three supported modalities, Eye2Gene applies the ensemble model corresponding to the modality of the scan to obtain a single scan-level gene prediction. Given multiple scans from a single patient over one or more appointments, Eye2Gene is applied to each scan in turn and the resulting predictions are combined to produce a single prediction for the patient, by taking the average over individual (postsoftmax) scan-level predictions per modality and then averaging across modalities (Fig. 1 and Supplementary Fig. 2).

### Eye2Gene generalizes across IRD clinics

To evaluate Eye2Gene, we simulated the scenario of applying Eye2Gene to retinal scans acquired over one or more appointments per patient in our internal MEH test set of 28,174 retinal scans from 524 patients from a held-out internal test dataset, as well as on a further external test dataset of 39,596 retinal scans from 836 patients from five different external IRD clinics, which included the Oxford Eye Hospital (UK), Liverpool University Hospital (UK), University Hospital Bonn (Germany), Tokyo Medical Center (Japan) and the Federal University of São Paulo (Brazil). For each patient we ran Eye2Gene on all scans to get an overall prediction per patient and compared the prediction of Eye2Gene to the underlying gene diagnosis.

Eye2Gene attained an overall top-five accuracy (the proportion of cases the correct gene appeared in the top-five ranked choices of the model) of 83.9% (81.7–86.0%) across patients in all test datasets. A breakdown of results per centre is shown in Table 1.

**Table 1:** Overview of Eye2Gene results on test data across different IRD clinics plus demographic characteristics

IRD clinics	Number of patients (number of images)	Number of unique genes	Median age	Percentage female	Ethnicity distribution				Anticipated top-five accuracy	Top-five accuracy
					Percentage white	Percentage Asian	Percentage Black	Percentage admixed		
Oxford	390 (29,145)	33	44.5	38	90	10			89.6%	90.1%
Liverpool	156 (6,174)	27	30	55	89	10		1	87.9%	88.2%
Bonn	129 (2,838)	12	43.7	48	85			15	90.4%	87.6%
Tokyo	60 (1,493)	24	29	54	2	97		1	71.0%	70.4%
São Paulo	40 (1,494)	10	35	55	45			55	89.1%	93.9%
**All external**	**775 (39,854)**	**42**	**39.7**	**47**	**66**	**30**		**4**	**87.9%**	**87.9%**
Moorfields	524 (28,174)	63	39	44	50	18	3	29	**–**	77.8%
**All test data**	**1,299 (68,028)**	**63**	**39.4**	**46**	**59**	**25**	**1**	**14**	**–**	**83.9%**

Top-five accuracy represents the proportion of case for which the correct gene appeared in the top-five ranked choices of the model. Anticipated top-five accuracy refers to extrapolated accuracy based on per-gene accuracy at MEH extrapolated to target dataset gene distribution. Note that unspecified ethnicity is not accounted in the ethnicity distribution reported here. Bold indicates summary data across sites.

Both ensembling across multiple networks and ensembling across images and/or modalities is crucial to Eye2Gene performance. Mean per-network top-five accuracy percentages were 68.9, 70.8 and 74.9% for FAF, IR and OCT, respectively, which improved to 71.0, 72.7 and 77.2% after ensembling across networks. In both the ensembled (83.9%) and unensembled (81.5%) case, combining predictions across multiple images and modalities led to higher accuracy than the best performing single modality.

Along with top-five accuracy, a commonly used metric in the literature for large multiclass problems^20–23^, we also applied more flexible conformal prediction sets that dynamically selects a number of predictions according to a desired accuracy threshold^24^. Selecting the 90% threshold we found a mean prediction set size of 8.1 genes with an empirical coverage (accuracy) of 90.5% (Extended Data Table 1). We also tested whether Eye2Gene’s accuracy was biased based on ethnicity or demographic parameters such as age and sex. A slightly lower performance was found in the Asian ethnic group, however, none of the differences were statistically significant in our test set (Supplementary Fig. 3). No statistically significant differences in accuracy on the basis of age and sex were found either.

### Eye2Gene predictions outperforms human expert-level accuracy

To contextualize Eye2Gene’s performance at image interpretation relative to clinical specialists, we asked eight ophthalmologists specializing in IRDs, with 5–15 years of experience, to predict the causative gene based on a single FAF image per patient across 50 different patients from the internal held-out test set. In this task, ophthalmologists were asked to identify the correct diagnostic gene from 36 provided (instead of Eye2Gene’s 63) when shown an FAF scan of a patient with an IRD. Historically, FAF has been a widely used imaging modality for the characterization of IRDs and hence a modality that many specialist ophthalmologists are likely to be the most familiar with using when diagnosing IRDs^25,26^. On this task the ophthalmologists achieved an average top-five accuracy of 29.5%, compared to 76% for the Eye2Gene module trained on FAF images only when applied to the same images (restricted to single-image predictions and only using the five-network FAF ensemble for a fair comparison). The results of the human benchmarking by ophthalmologists are presented in Table 2. As expected, human performance tended to improve with the level of experience but the performance of Eye2Gene was considerably better than any single human expert.

**Table 2:** Benchmarking Eye2Gene against humans

Ophthalmologist	Years of experience specializing in IRDs	Correct top-five guesses
1	5	13 (26%)
2	5	14 (28%)
3	5	14 (28%)
4	6	15 (30%)
5	7	13 (26%)
6	10	15 (30%)
7	15	16 (32%)
8	15	18 (36%)
**Ophthalmologists**’ **average**		**14.75 (29.5%)**
Eye2Gene		38 (76%)

For comparison, eight ophthalmologists and Eye2Gene classified 50 FAF retinal scans of 50 patients results from the MEH internal held-out test dataset. Bold indicates summary data across sites.

### Eye2Gene for phenotype-driven genetic variant prioritization

**Fig. 2:**
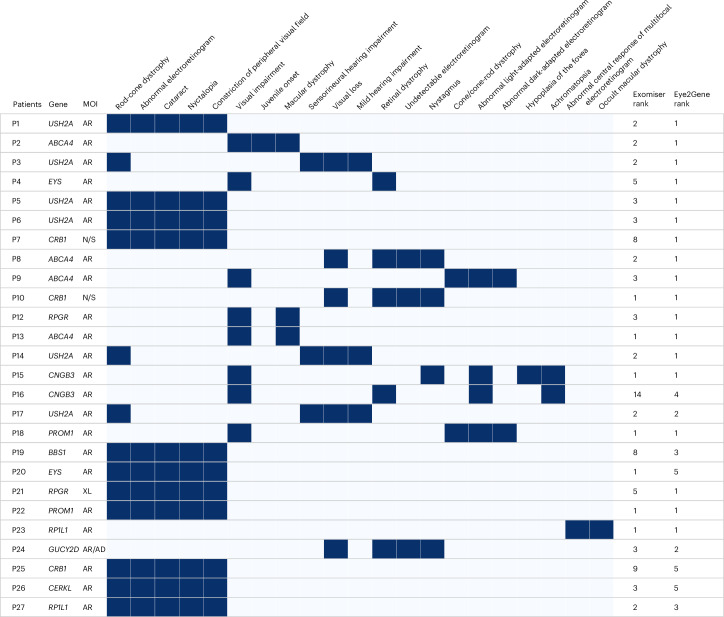
Eye2Gene for gene prioritization. Phenotype grid for sample of 27 from 130 individuals with IRD for which the Exomiser-hiPHIVE gene rank based on HPO-only was compared to the Eye2Gene gene rank based on retinal scans. Eye2Gene outranks the HPO-only approach in 75% of the cases. Each row represents a patient. Each column represents an HPO term. Dark blue cells represent presence of HPO term and light blue represent absence. MOI indicates mode of inheritance, which can be autosomal recessive (AR), autosomal dominant (AD) or X-linked (XL). Note that some HPO terms are not retinal specific such as sensorineural hearing impairment and mild hearing impairment. N/S, not specified.

Genetic variant prioritization is an important task to diagnose single-gene conditions such as IRDs especially given the large number of genetic variants reported by whole genome sequencing^27,28^. We evaluated the use of Eye2Gene in aiding genetic variant prioritization. Clinical notes and retinal scans of 130 individuals with IRD with confirmed gene diagnosis, who were part of the Eye2Gene test set, were reviewed and 21 distinct human phenotype ontology (HPO) terms were identified (4.7 on average per patient). Each patient’s HPO terms were used as input for the Exomiser-hiPHIVE phenotype-gene scoring algorithm^29^. Each patient’s retinal scans were used as input for the Eye2Gene phenotype-gene score. We found that Eye2Gene provided a rank for the correct gene higher or equal to the Exomiser-hiPHIVE score in more than 75% of the patients (Wilcoxon rank sum *P* < 5.0^−10^), showing that image-based gene predictions can outperform HPO-only predictions for IRDs even when those include non-retinal specific HPO terms such as ‘Sensorineural hearing impairment’ and ‘Mild hearing impairment’ (Fig. 2).

### Eye2Gene identifies new gene–phenotype groupings

**Fig. 3:**
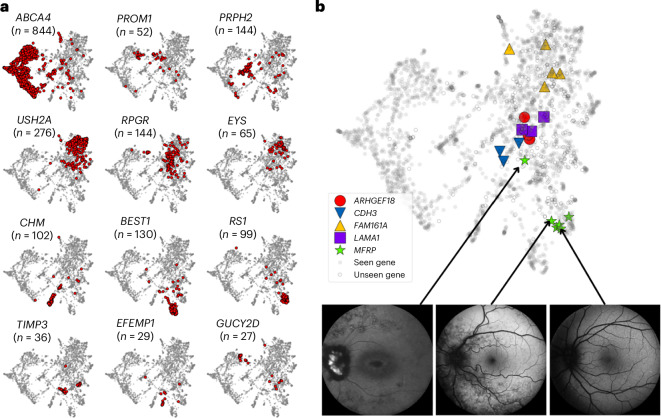
Visualization of Eye2Gene embeddings. **a**, Two-dimensional visualization of the embeddings obtained from Eye2Gene for select genes. Each point corresponds to an individual FAF scan. Each point in red represents a scan from a patient with the corresponding gene. A total of 170 unique genes are represented, a full list is included in the supplementary materials (Supplementary Fig. 6). These 2D embeddings are obtained by applying the UMAP dimensionality reduction algorithm to the penultimate layer of Eye2Gene, a 768-dimensional vector. **b**, Embeddings for five unseen genes. Solid circles represent individuals with a gene diagnosis for one of the 63 genes that Eye2Gene was trained on, hollow circles represent individuals from other ‘unseen’ genes. Five exemplar genes, not included in the Eye2Gene training dataset of 63 genes, are highlighted by the different symbols.

We evaluated the use of Eye2Gene as a next-generation phenotyping tool to identify new gene–phenotype groupings. We extracted the activations from the penultimate layer of the FAF component of Eye2Gene to obtain high-dimensional latent embeddings of each FAF scan. The uniform manifold approximation and projection (UMAP)^30^ dimensionality reduction algorithm was applied to the extracted activations to obtain two-dimensional representations for visualization purposes (Fig. 3a). Patients with the same genetic diagnosis frequently cluster in embedding space, even in the case of genes never encountered by Eye2Gene such as *ARHGEF18* and *CDH3* (Fig. 3b). As well as where patients cluster together, outliers can also be informative. For example, the outlying *MFRP* patient in Fig. 3b has hyper-autofluorescent optic disc drusen not present in the other individuals with *MFRP*. These observations could be useful for highlighting specific phenotypic subgroups associated with certain genes, linked to specific inheritance patterns (for example, *BEST1* associated with autosomal dominant Best disease and autosomal recessive bestrophinopathy^31^). Additionally, this could assist in identifying potential misdiagnoses or individuals with dual pathology. These embeddings also matched known phenotypic associations, for example genes associated with Retinitis Pigmentosa (for example *USH2A*, *RPGR*, *EYS*) cluster top right, while cone-rod associated *ABCA4* appears overwhelmingly on the left, with phenotypically similar *PRPH2* and *PROM1* occupying the space in between.

We also found that individuals within the same family group tended to cluster closer together (Supplementary Fig. 4), with a mean inter-patient Euclidean distance of 41.3 in embedding space for individuals within the same family group, compared to 58.9 for a non-parametric bootstrap that resamples random pairs of patients (*P* < 10^−10^).

To see what genes tended to appear close to each other we applied hierarchical clustering to the raw embeddings (pre-UMAP) to produce a hierarchy of phenotypic similarities according to Eye2Gene embedding space. The resulting dendrogram (Supplementary Fig. 5) captures some of the known phenotypic similarities in IRDs such as Stargardt phenocopy genes (*ABCA4*, *PRHP2* and *PROM1*), retinitis pigmentosa genes (*RPGR* and *USH2A*) and achromatopsia genes (*CNGA3* and *CNGB3*). This shows that Eye2Gene’s data-driven approach has the potential to identify phenotypically similar genes, even on new gene classes not included in the training data (Supplementary Fig. 6).

### Eye2Gene distinguishes genetic from non-genetic disease

**Fig. 4:**
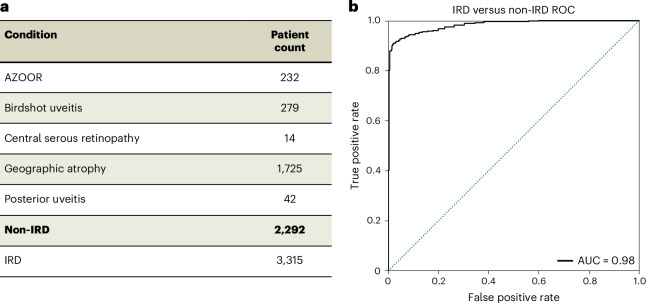
Eye2Gene as a screening model. **a**, Dataset including individuals with IRD and non-IRD. Individuals with a non-IRD were selected on the basis of having a condition with a similar retinal presentation in FAF. Bold indicates the total count of non-IRD individuals. AZOOR, acute zonal outer occult retinopathy. **b**, ROC classifier of a binary CoAtNet deep learning classifier trained on dataset **a**. The accuracy is 93.8% on the test set of 20% of the images, with an AUROC of 0.98. AUC, area under the curve.

As not every patient that presents to an IRD clinic necessarily has a genetic condition, an additional Eye2Gene module was developed as a screening tool to detect presence or absence of IRDs, to identify individuals with other non-IRD conditions. Given there are many retinal conditions that cause atrophy, it is often challenging to distinguish IRD from other non-monogenic disease aetiologies, such as age-related macular degeneration (which can mimic macular dystrophies and vice versa), inflammatory changes such as autoimmune retinopathies, posterior uveitis and acute zonal outer occult retinopathy, as well as certain drug toxicities (including retinopathies associated with hydroxychloroquine, pentosan polysulfate and antiretroviral medications). Eye2Gene can serve as a screening tool based on conditions with a similar presentation (differential diagnosis), providing an area under the receiver operating curve (AUROC) of 0.98 (Fig. 4).

### Eye2Gene predictions outperform other AI approaches

As well as comparing favourably against gene prioritization approaches based on HPO terms^21,32^, Eye2Gene outperforms previously published image-based AI approaches trained on smaller less diverse imaging datasets and for more limited classification tasks that distinguish up to four genes (Supplementary Table 1). For example, Miere et al.^33^ achieved an accuracy of 88% in distinguishing *ABCA4* from *PRPH2* based on FAF scans, whereas on the same task Eye2Gene performs 90.8% (Supplementary Table 1). Miere et al.^34^ also obtained an accuracy of 94.6% for three broad IRD phenotypes (retinitis pigmentosa, Best disease and Stargardt disease) from FAF images, compared to 95.6% for Eye2Gene on a similar task. Similarly Fujinami-Yokokawa et al achieved an accuracy of 89.3% on OCT^35^ and 94.6% on FAF^36^ in distinguishing *ABCA4*, *RP1L1* and *EYS*, whereas Eye2Gene, on the same task, achieves an accuracy of 96.3% on OCT and 96.3% on FAF (Supplementary Table 1). Shah et al.^37^ trained a classifier on SD-OCT to distinguish only one type of IRD (Stargardt) from normals and obtained an AUROC of 0.99 whereas Eye2Gene is able to distinguish any IRD out of 189 from non-genetic conditions with a similar phenotype with an AUROC of 0.98 (Supplementary Table 1). Although Eye2Gene, by definition, is not able nor designed to outperform genetic testing, as it trained on gene labels derived from genetic testing, we have demonstrated its utility in guiding the prescription and interpretation of genetic testing by AI-powered phenotyping of retinal scans.

### Eye2Gene is accessible as an online web application

**Extended Data Fig. 1:**
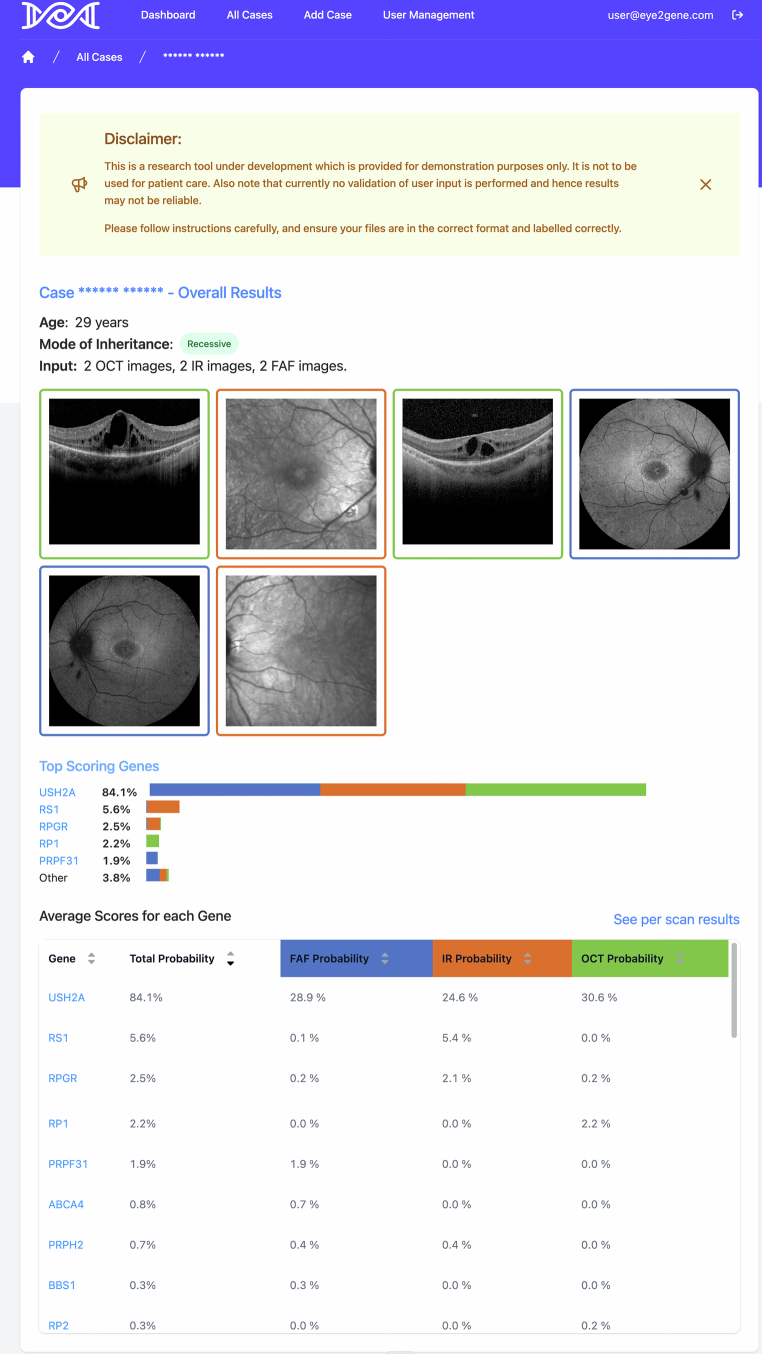
Output of Eye2Gene app on an example case of a patient with disease-causing variants in the *USH2A* gene. The user is presented with a bar chart of the top 5 genes as predicted by the Eye2Gene model, along with the model probability score for each gene. These predictions are broken down into the contributions of the three different modalities, which are displayed as different colors on the bar-graph (blue for fundus autofluorescence, orange for infrared and green for optical coherence tomography). The input images, color-coded by modality, and patient information are included at the top of the display. A full breakdown, with predicted probabilities for all 63 genes, is presented in the table below the bar graph. A link in the top right of the table takes the user to a breakdown of Eye2Gene’s predictions for each of the uploaded images.

To demonstrate how Eye2Gene could be used to assist in diagnosing individuals with IRD, Eye2Gene is accessible as a web application online at https://app.eye2gene.com to be used as a research tool. Users upload a series of scans of the supported modalities (SD-OCT, 55-degree FAF and 30-degree IR) from a single patient. These images are then passed to the Eye2Gene model, which outputs a set of prediction scores for each of the 63 genes on each of the input scans. This information is aggregated into an overall Eye2Gene prediction score for that case and presented to the user (Extended Data Fig. 1). This score can be embedded in genetic variant prioritization frameworks.

## Discussion

We present Eye2Gene, a deep learning model for classifying 63 causative genes in individuals with IRDs, using retinal scans acquired using three different imaging modalities. These scans can be obtained via non-invasive eye scans using widely available technology. We have comprehensively evaluated Eye2Gene on internal datasets, against human experts and validated it on external datasets. We have shown that Eye2Gene can greatly improve the phenotyping capabilities required in the genetic diagnosis of IRDs.

So far, only four previous studies have tried to apply AI to IRDs, all in much smaller datasets of fewer than 150 patients and across substantially fewer genes (Supplementary Table 1). By comparison, Eye2Gene, due to the benefit of having been trained on one of the world’s largest datasets of genotyped individuals with IRD (*n* = 2,451), has potential for much broader utility, as it supports as many as 63 gene diagnoses, multiple imaging modalities and has conducted external validation.

In clinical settings, where decisions directly affect patient care, interpretability of AI decision support systems in terms of both the input images as interpreted by expert users and the output probabilities as interpreted by all users is essential for validating model outputs and building trust with patients and healthcare providers. Eye2Gene currently provides interpretability of input images (1) by attention maps (Supplementary Fig. 7) and (2) by visualization of cases in embedding space to identify phenotypically similar cases (Supplementary Fig. 8), and of output probabilities (3) by uncertainty estimates through conformal predictions (Supplementary Table 2 and Supplementary Fig. 9). In most applications, we anticipate Eye2Gene will be used with clinician oversight and backed up with genetic testing (where available), reducing the associated risks.

Of course, Eye2Gene predictions will not replace the need for genetic testing or counselling at specialized IRD centres, especially when a treatment such as a gene therapy is to be administered on the basis of a confirmed genetic diagnosis. Furthermore, in some contexts using more transparent approaches may be required, for example when conducting longitudinal analysis that Eye2Gene is not designed for. In these instances, quantitative analysis of biomarkers with conventional statistical approaches, for example using AI segmented imaging features via AIRDetect^38^, may be preferable to ‘black box’ deep learning-based approaches such as Eye2Gene. Therefore, currently, the main anticipated application of Eye2Gene is in facilitating more efficient cost-effective genetic diagnosis by indicating when molecular testing would be worthy of consideration, and guiding specific genetic testing and/or its interpretation (Supplementary Fig. 10). It could also open the door to point-of-care identification of potential causative genes especially in geographic regions where genetic testing is not available^39^.

Human expert benchmarking showed that the task of identifying likely genes from retinal phenotypes is very challenging even for IRD clinical experts with many years of experience. However, retinal phenotyping is a task that is often required of clinician experts, as they either need to provide input as to whether a patient with a retinal condition should undergo genetic testing, or whether the retinal phenotype is in keeping with a specific genetic condition when interpreting the results of a genetic test as part of multidisciplinary team meetings^40^. Additionally, the process of gene discovery involves identifying other individuals with similar retinal phenotypes. We have shown that the Eye2Gene AI is very adept at these tasks.

Eye2Gene gene predictions aid the interpretation of the results of a multigene genetic test by scoring and thereby prioritize genetic variants where the gene matches the phenotype^21^. In disorders with a highly distinct phenotype, the matching phenotype PP4 criterion in the American College of Medical Genetics guidelines provides a higher level of evidence in variant classification when the phenotype is consistent with the genetic aetiology, which might be crucial for the transition from a variant of unknown significance to a likely pathogenic variant. This approach has been shown to improve diagnostic yield for other highly heterogeneous disorders such as syndromic intellectual disability and can improve the diagnostic yield in other pipelines where over 10% of the variants are of unknown significance^8^. Using next-generation phenotyping approaches such as Eye2Gene, GestaltMatcher^23^ and Deeplasia^41^ for monogenic conditions it is now possible to incorporate PP4 into variant interpretation in an objective way, which could increase the diagnostic yield for these conditions without relying on phenotypic labels such as HPO terms that can be ambiguous and also increase the risk of unwanted incidental findings in genomic pipelines.

Eye2Gene is an AI algorithm that demonstrates strong potential for clinical use in the challenging area of IRDs. Despite these promising results, we recognize that are still several limitations to Eye2Gene and it remains in active development to address and alleviate these issues.

First, Eye2Gene accuracy is affected by the gene distribution of the target dataset (Supplementary Fig. 11), which we show in Table 1 accounts for most of the variability seen in Eye2Gene accuracy on the basis of the anticipated top-five accuracy across external sites. While the Eye2Gene development and test datasets are likely to closely match the underlying gene distribution of the underlying patient population of London, UK, where MEH is based, gene distributions can vary between different patient populations and across patients of different ethnic backgrounds (Supplementary Fig. 12). For example, Eye2Gene appears to perform slightly worse on patients from Asian and South Asian backgrounds (although not statistically significant) (Supplementary Fig. 3). This highlights the need to include diverse training datasets that are sufficiently representative of the target patient population(s), and avoid widening existing health disparities^42^. In the future we plan to include data collected from more global sites into our training data, particularly those with large patient populations from non-European ethnic backgrounds such as from Asia and Africa. To this end strategies such as federated learning may be critical in reducing barriers surrounding sharing of data^43^. We are also considering other methodological approaches for mitigating dataset bias such as data augmentation with generative AI and nearest matches based approaches for rare classes^22,44^. Nonetheless in spite of dataset limitations, as it stands, Eye2Gene performance is within the anticipated accuracy (<5%) as extrapolated from the MEH training dataset to the gene distribution of the different centres. Furthermore, Eye2Gene already generalizes well as the top-five accuracy is consistently above 70% even in the ethnically distinct Tokyo site where the ethnicity of patients is predominantly Asian (Table 1). We also did not find any statistically significant evidence of sex or age bias in Eye2Gene’s accuracy, which suggests the bias is unlikely to be large. Eye2Gene’s ability to generalize is in part due to the MEH training dataset being primarily based in London, which represents an ethnically diverse population that receives patients from all over the UK (Supplementary Table 2 and Supplementary Fig. 13). Although we did not identify any large systematic difference in terms of image quality between the sites (Extended Data Table 3), accuracy is known be linked to image quality^45^ (Supplementary Fig. 14). In future work it may also prove useful to do a more in-depth analysis of how image acquisition parameters may influence Eye2Gene results.

Second, Eye2Gene is currently limited to predicting 63 gene classes out of a potential of 281 genes that are currently known to be associated with IRDs, not all of which are present in the Moorfields dataset (Supplementary Fig. 15), and hence cannot be used to predict IRDs that are not in those 63. However, we found that Eye2Gene embeddings were effective in identifying phenotypically similar cases, even for genetic conditions that Eye2Gene was not trained to identify (Fig. 3). In the future, by leveraging these embeddings, Eye2Gene could be extended to identify additional or similar IRDs, for example through a matching approach similar to GestaltMatcher^22^, which could also have positive effects for accuracy on rare genes.

Third, the comparison with experts in the present study was based on images only. We acknowledge that although this task does not capture the full scope of standard clinical practice as clinical decisions include other clinical parameters, it demonstrates the strength of Eye2Gene and its potential in assisting even IRD specialists in tasks such as genetic results interpretation that could include the secondary analysis exploration of the genetic data for less obvious genetic candidates such as non-coding or structural variants.

Future comparisons could also incorporate additional clinical information, however, our experiment demonstrates that Eye2Gene already has a role as an adjunct tool that experts could use in multidisciplinary team meetings to assist image interpretation^23^.

In conclusion, Eye2Gene shows that next-generation phenotyping using AI is a promising approach to aid in the genetic diagnosis for individuals with IRDs, something that is not only important for improving patient experience and reducing associated overheads, but is likely to become especially important due to the growing number of potentially treatable IRDs where a rapid genetic diagnosis can lead to an improved outcome for the patient^46^.

## Methods

### Dataset quality control and preparation

The MEH IRD cohort was previously described by Pontikos et al.^1^ and encompasses 4,501 individuals with IRDs caused by variants in 189 distinct genes, of which 324 individuals (with variants in 72 genes) were younger than 18 years of age as of 2 August 2019. Individuals with an IRD and a confirmed genetic diagnosis by an accredited genetic diagnosis laboratory were identified and information about the genetic diagnosis was exported from the MEH electronic health record (OpenEyes) using a SQL query on the Microsoft SQL Server hospital data warehouse database.

Images were exported from the MEH Heidelberg Imaging (Heyex) database (Heidelberg Engineering) for all individuals with an IRD, on the basis of their hospital number, for records between 25 March 2004 and 22 October 2019. We selected the Heidelberg Spectralis as it is one of the most widely used medical imaging devices in IRD clinics worldwide and has previously been applied to AI-based approaches on IRDs. This resulted in a dataset of 2,103,692 images from 264,299 scans in 4,510 patients. For the quality control and data preparation process, images were divided by modality, with 87,534 short-wavelength FAF images in 4,000 patients, 35,608 IR images in 3,731 patients and 1,647,349 SD-OCT images in 3,731 patients. Since SD-OCT produces several B-scans, for each SD-OCT volume we selected only the median four B-scans corresponding to the four scans closest to the scan that traverses the fovea, as they were likely to be the most informative. Following this, 141,895 SD-OCT B-scans remained in 3,728 patients.

For all three modalities, we applied the filtering as shown in Supplementary Fig. 16. Any corrupted (unreadable) images were discarded. FAF scans feature two different imaging magnification levels, 30 degrees and 55 degrees. We kept 55-degree images and all other images were discarded, using data from the scan metadata to distinguish the two cases.

To remove low-quality and defective images, we used Retinograd-AI model to filter out poor-quality scans^47^. These models were applied to the all FAF and SD-OCT images (using only the median B-scan for SD-OCT) within our dataset to obtain a prediction for each, and then all scans with a gradeability score of partially and un-gradable were rejected. Since IR and SD-OCT scans are captured simultaneously, for IR scans we took the gradeability score of the corresponding SD-OCT volume as the label and filtered similarly. After this process, 27,433 FAF, 33,706 IR and 134,293 (33,712 volumes) SD-OCT images remained in 3,315, 3,715 and 3,715 patients, respectively.

The number of images rejected at each stage of the process is provided in Supplementary Fig. 16. To ensure sufficient data for training and testing, we restricted our datasets to only genes with at least ten patients remaining after filtering, leaving 63 individual genes. The distribution of all 63 genes is presented in Supplementary Fig. 10 and the full breakdown by gene is given in Extended Data Table 2. Following the quality control and gene selection process, 25,233 FAF, 31,357 IR and 124,975 (31,363 volumes) SD-OCT images remained across 3,652 patients in 63 distinct genes. The phenotype distribution of a subset of 2,103 of these patients is provided in Supplementary Table 4 per gene and per phenotype in Supplementary Table 5. The visual acuity distribution across genes is provided in Supplementary Fig. 17.

Postquality control, these patients were split into a ‘development’ set of 3,128 patients, and a held-out internal test set of 524 patients (28,174 images). Stratified sampling was used to ensure at least three representative patients for each gene were present in the test set, and to ensure no families were present in both test and development sets. The development set was further split into train and validation sets according to an approximate 80/20 spit, with 2,451 patients (119,755 images) in the training set and 677 patients (31,605 images) in the validation set. The training set was used to train our 15 constituent Eye2Gene networks, while the internal test set was kept separate to enable testing of the final Eye2Gene model.

In addition to the MEH data described above we also obtained images from a further five centres to enable external validation of Eye2Gene. Oxford Eye Hospital (UK) provided a sample of 29,145 scans from 390 patients with distinct gene diagnoses in 33 different genes. The University Eye Hospital of Liverpool (UK) provided a sample of 6,174 scans from 156 patients with distinct gene diagnoses in 27 different genes. The University Eye Hospital Bonn (Germany) provided a sample of 2,838 scans from 129 patients with distinct gene diagnoses in 12 different genes. The Tokyo Medical Center (Japan) provided a sample of 1,493 scans from 60 patients with distinct gene diagnoses in 24 different genes. The Federal University of Sao Paulo (Brazil) provided a sample of 1,494 scans from 40 patients with distinct gene diagnoses in ten different genes. The MEH (UK) internal test dataset consisted of 28,174 scans from 524 patients across 63 gene diagnoses. Further breakdown by dataset is available in Extended Data Table 3 and with further breakdown by gene in Supplementary Table 6. Retrospective images from patients were selected by the clinical team at each of the five external centres according to the requirements: (1) that the patients had a confirmed genetic diagnosis within one of the 63 genes that are currently recognized by Eye2Gene; (2) the patient had retrospective retinal imaging available that was 55-degree FAF images or 30-degree OCT images obtained from the Heidelberg Spectralis as part of routine care and (3) the retinal images were considered of good quality. No further requirements were given regarding the ethnicity or the sex of the cases. For each patient a set of scans was selected, typically consisting of one scan per-patient per-eye per modality, along with their genetic diagnosis. These data were shared with us through our secure online portal, with the exception of the data from Bonn where the Eye2Gene models were run locally. No preprocessing or standardization of the retinal images took place but the image quality computed using Retinograd-AI^47^ confirmed that the images were of comparable quality between centres and of slightly higher quality overall than those in the MEH test set given those ones were not explicitly selected by a clinician (Extended Data Table 3).

### Model training

On each modality, five 63-class CoAtNets were trained for 100 epochs (passes over the entire dataset) for FAF and IR, and 25 epochs for OCT, which was found to be sufficient for the validation accuracy to converge in most settings (Supplementary Fig. 18). Random initialization with different random seeds was used for each individual of the 15 networks to ensure ensemble diversity. A CoAtNet0 architecture from the keras-cv-attention-models pypi library was used, where the final output layer was replaced by a dropout layer, followed by a linear layer with 63 outputs and softmax normalization. The CoAtNet architecture was chosen on the basis of an initial comparison of a number of different architectures evaluated on the FAF dataset. Cross entropy loss was used for the loss function, using additional class-weighting inversely proportional to gene frequency in the dataset, where the labels were given by the gene diagnosis of the underlying patients. This was to help address dataset imbalance due to the non-uniform gene distribution. For training, the Adam optimizer was used with the default parameters used in the Keras library (*β*_1_ = 0.9, *β*_2_ = 0.999). Learning rate was set to 0.0001 as it was found to work well across a range of architectures, and the batch size was set to 16, as this was the largest we could fit in graphical processing unit (GPU) memory. Dropout probability was fixed at 50%. Training was completed within 8 h for each neural network training on a single 3090 24 GB GPU.

To avoid overfitting to the training data, data augmentation techniques were applied. A number of plausible image transformations were applied automatically to the input data during training (Supplementary Fig. 19).

### Evaluation

The output prediction of Eye2Gene is obtained by combining the predictions of its 15 constituent networks using an ensemble approach. For each single retinal scan of a specific modality, a five-model ensemble is applied by taking the simple arithmetic mean of each of the output probabilities per gene across the five constituent networks. Given a collection of retinal scans from a single patient, the appropriate ensemble model corresponding to each scan’s modality is applied, then the average predictions across all scans within each modality in the collection is taken in the same manner for a per-modality prediction per patient. Finally, the average across the three modalities is calculated and used as the final prediction for the patient. This approach was applied across all available scans per patient. Although there may be individual cases where it is better to down-weight or exclude certain images, or modalities overall we find that including more images per patient improves the overall accuracy (Supplementary Fig. 20). Additionally, we experimented with different class weightings, performing a grid search over modality weightings in 0.1 increments from 0 to 1.0 on the development validation set (the weights do not need to sum to 1 as only the relative class predictions affect the prediction). This improved validation top-five accuracy from 82.6 to 84.0%, with weights of 0.8, 0.1 and 0.5 for FAF, IR and OCT; however, applying this same weighting to the test data led to decreased top-five accuracy from 83.9 to 83.5%. With sufficient calibration data it may be possible to more accurately determine the optimal modality weighting; however, in the absence of other evidence, equal weighting provided a sufficiently good heuristic.

The model predictions were then compared against the underlying gene diagnoses for each patient to compute the overall accuracy of the model on the test data, the top-*k* accuracy (the proportion of images where the correct gene was within the highest *k* predictions of the network) for *k* = 2,3,5,10, and the average per-class F1, weighted F1, mean average precision (MAP) and AUROC (Supplementary Table 7). Accuracy was calculated by counting the number of times Eye2Gene’s top prediction matched the gene of the underlying patient. Per-gene precision-recall curves (for MAP) and ROC were produced for each gene in a one-versus-rest setup, using the Eye2Gene predictions for each output gene, and areas under the respective curves were calculated using trapezoid estimation (Supplementary Fig. 21). Confidence intervals were obtained by bootstrapping over 10,000 resamplings and taking the 2.5th and 97.5th percentiles. For convenience, all predictions were compiled into a single .CSV file along with additional data about each image (such as patient study ID, gene, appointment date) and the ID of the model used to generate the prediction. Eye2Gene combines predictions across multiple models (ensembling) and across multiple images acquired during one or more patient visits.

Taking each network individually without ensembling, the mean per-network top-five accuracies per image were 68.9, 70.8 and 74.9% for FAF, IR and OCT on our full validation dataset (which includes external sites). Applying ensembling of the five models per modality to the individual images we observed accuracies of 71.0, 72.7 and 77.2% for FAF, IR and OCT. Combining individual model predictions across multiple images (without ensembling the five models per modality) at the per-patient level, on the held-out test set, we observed an overall mean top-five accuracy across models of 81.5%. In general, we found that combining all images across all three modalities typically outperformed the best performing single modality (that is restricting to images of that modality only) on most genes, demonstrating the advantage of the multi-modality approach. In both cases these were superior to the single-network results, but inferior to the overall Eye2Gene model (83.9%), suggesting that both using ensembling across networks at a per-image level, and ensembling predictions across multiple images, was advantageous.

### Conformal prediction

Conformal prediction sets construct a set of candidate classes instead of single class outputs. Crucially, this set is not fixed in size but is dynamically sized to reach some user-defined confidence threshold. This means that for ‘easy’ examples, the prediction set may be very small (or even just one class) but allows for larger prediction sets for more ambiguous examples. By adjusting the confidence threshold, we can trade off between the proportion of example instances in which the correct class was in the prediction set, which is defined as coverage, and the set size. Conformal prediction sets can be constructed for any classifier with probability outputs and are useful tool for interpretability of model output probabilities. The basic ‘naive’ conformal set construction algorithm is fairly simple and just increases the predicted classes included in the conformal prediction set until the desired confidence threshold is met; however, model outputs are often poorly calibrated in practice. Hence, various algorithms exist to calibrate conformal prediction sets (Supplementary Table 2).

We apply the least ambiguous adaptive prediction sets conformal prediction method, taking per-class probability outputs from Eye2Gene and adding the classes to the prediction set until the predetermined confidence threshold was exceeded. We selected least ambiguous adaptive prediction sets from among three methods, as it produced smaller average prediction sets for a given coverage value, which we report in Supplementary Fig. 9. We calibrate our conformal prediction confidence levels on the MEH model validation set (*n* = 677) (not seen by the model during training) taking the compiled predictions for each patient.

### Evaluating phenotype-driven genetic variant prioritization

Clinical notes and retinal scans of 130 patients with IRD from MEH with a confirmed gene diagnosis, who were part of the Eye2Gene test set, were manually reviewed and HPO terms were identified by three ophthalmologists with expertise in IRDs as described in Cipriani et al.^21^. These HPO terms were used as input for the latest version of the Exomiser-hiPHIVE algorithm (v.14.0.0) (https://github.com/exomiser/Exomiser) to obtain a gene ranking for the most probably predicted gene. The Exomiser-hiPHIVE algorithm uses a gene-specific phenotype score based on the PhenoDigm algorithm between the patient’s phenotype encoded as a set of HPO terms and the phenotypic annotation of any known gene-associated phenotypes reported in disease databases that include human and model organisms such as mouse and zebra fish. The retinal scans for these 130 patients with IRD were also analysed using Eye2Gene to obtain gene predictions that were also ranked accordingly for direct comparison with the Exomiser-hiPHIVE ranking. The non-parametric Wilcoxon rank sum test was used to compare the Exomiser-hiPHIVE and Eye2Gene gene rankings. Note that the Exomiser also takes as input the genetic variants file (.VCF file), which also gives it an advantage over Eye2Gene in terms of reducing the set of possible genes to consider only genes that contain genetic variants that may be considered pathogenic.

### Visualization and clustering of model embeddings

Visualizing and clustering model embeddings are valuable data-driven approaches for evaluating class diversity and identifying similarity between classes, even for new classes that the model has not been trained on. We applied one of the Eye2Gene FAF networks to all FAF scans in our test dataset, and the activations of the penultimate hidden layer were extracted to give a 768-dimensional vector for each scan. The UMAP dimensionality reduction algorithm was applied to the extracted activations, to obtain two-dimensional embeddings. The embeddings in UMAP space were then clustered using hierarchical clustering with Ward linkage, to produce a gene groupings dendrogram. By visualizing the UMAP-projected embeddings of the retinal images obtained from the different centres, we were able to show that no centre clustered separately and hence the images are unlikely to be systematically different (Supplementary Fig. 22). Using the embeddings, we were also able to derive a prototype-based methods inspired approach that matches the most similar images in embedding space according to the cosine similarity (Supplementary Fig. 8).

### Eye2Gene screening component

For the screening component of Eye2Gene, a neural network was trained to distinguish FAF images of patients with IRD from patients with non-IRD. Hyperparameters, network architecture and training settings were the same as for the main Eye2Gene FAF module, except no ensembling across models or images was used. For the patients with non-IRD, a number of conditions were selected for presentation similar to IRDs in FAF imaging: acute zonal outer occult retinopathy, birdshot uveitis, central serous retinopathy, geographic atrophy and posterior uveitis. Patients with these conditions were extracted from the MEH hospital database and processed in the same manner as the IRD data (*n* = 2,292). Patients with IRD, before filtering for genes with more than one case, were selected (*n* = 3,315) (Supplementary Fig. 15). For evaluation, a held-out test set of 20% of patients was kept, and used as an evaluation set. For plotting of the ROC curve and area under the curve calculation, the outputs of the network were treated as a binary classification by taking the output probability of the IRD class as the predictive probability.

### Interpretability of Eye2Gene image classifications

A well-known limitation of deep learning models in general is that their interpretability currently remains challenging and that existing approaches such as gradient-based saliency maps are not, as of now, sufficiently reliable for medical decisions. We leveraged the fact that the CoAtNet architecture uses self-attention to extract attention maps from one of the constituent Eye2Gene networks on a selection of FAF images. These attention maps show the areas of the image with the highest attention weights under the network’s self-attention mechanism, and hence the areas that are most strongly incorporated into the network’s final prediction (Supplementary Fig. 7). These maps were promising, consistently attending to areas of pathology, which are likely to be indicative of a particular condition according to our evaluation by human experts (Supplementary Fig. 22).

### Human benchmarking

To contextualize the performance of Eye2Gene compared to human experts, a challenge dataset of 50 FAF images from patients sampled from the MEH held-out set was created, with 36 unique genes, and no more than two of any given gene. FAF was selected since it is one of the imaging modalities most commonly used in IRD clinics and hence the one for which ophthalmologists should overall have the most experience in the assessment of IRDs. We asked eight ophthalmologists from Moorfields (M.M., A.R.W., O.A.M.), Bonn (F.G.H., P.H., B.L.), Oxford (S.R.D.S.) and Liverpool (S.M.), specializing in IRDs, with 5–15 years of experience, to predict the causative gene based only on the images provided. These eight ophthalmologists (M.M., A.R.W., O.A.M., F.G.H., P.H., B.L., S.R.D.S., S.M.) were selected on the basis of the criteria of being board certified specialists in ophthalmic genetics who run dedicated IRD clinics at their respective hospitals and would have reviewed on average retinal images from hundreds of patients per year. For each image, each ophthalmologist was asked to name the five genes they thought was most likely out of a list of 36 genes. Eye2Gene was then run on the same images, taking the top-five predictions from the full 63 genes that Eye2Gene was trained on and then compared to the clinicians’ predictions.

### Ethics

This research was approved by the Institutional Review Board and the UK Health Research Authority Research (HRA) Ethics Committee (REC) reference (22/WA/0049) ‘Eye2Gene: accelerating the diagnosis of inherited retinal diseases’ Integrated Research Application System (project ID 242050). The study sponsor was the University College London Joint Research Office (UCL JRO). The UCL JRO Data Protection reference number is Z6364106/2021/11/67. A summary of the research study can be found on the HRA website (https://www.hra.nhs.uk/planning-and-improving-research/application-summaries/research-summaries/eye2gene-10/). The REC that approved this study is Wales REC 5 (Wales.REC5@Wales.nhs.uk). All research adhered to the tenets of the Declaration of Helsinki.

### Reporting summary

Further information on research design is available in the Nature Portfolio Reporting Summary linked to this article.

## Supplementary information

Supplementary Information Supplementary Figs. 1–23 and Tables 1–7.

Reporting Summary

## Figures and Tables

**Extended Data Table 1:**
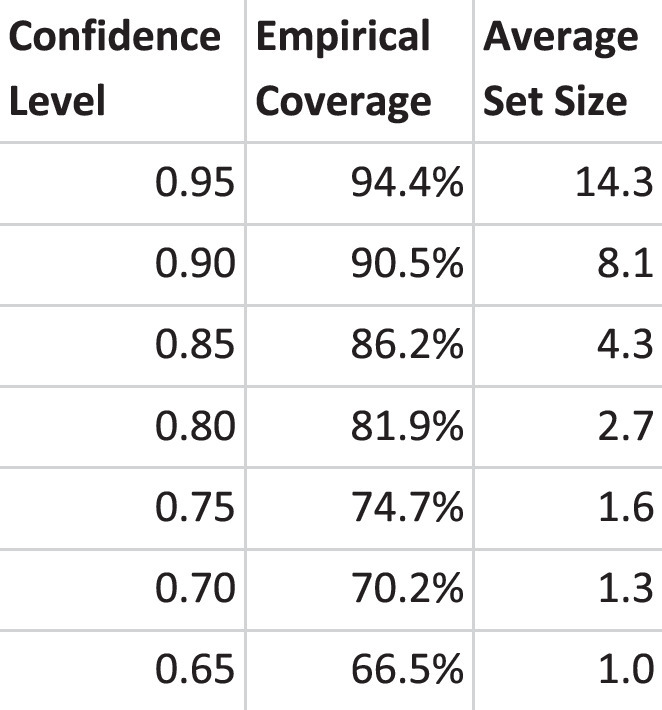
Overview of Eye2Gene conformal prediction set sizes at different confidence levels

Overview of Eye2Gene conformal prediction set sizes at different confidence levels.

**Extended Data Table 2:**
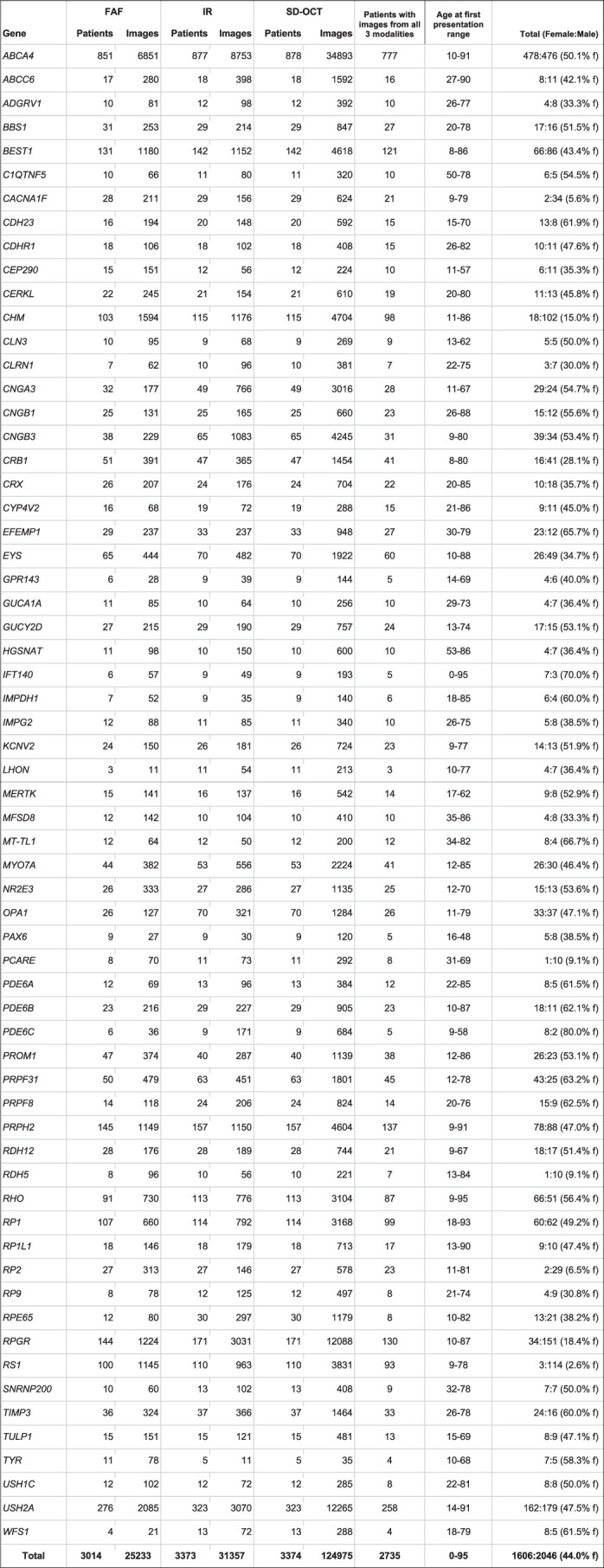
Number of patients and images per gene and modality (FAF, IR and SD-OCT) for the 63 genes included in the full Moorfields Eye2Gene development dataset post-QC (n = 3,652)

Number of patients and images per gene and modality (FAF, IR and SD-OCT) for the 63 genes included in the full Moorfields Eye2Gene development dataset post-QC (n = 3,652).

**Extended Data Table 3:**
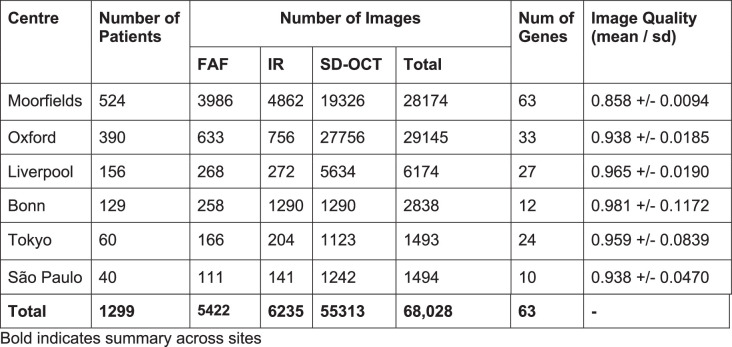
Number of patients, images and genes in internal (Moorfields) and external (Oxford, Liverpool, Bonn, Tokyo, and São Paulo) test datasets. Last column describes the image quality as calculated by Retinograd-AI^47^

Number of patients, images and genes in internal (Moorfields) and external (Oxford, Liverpool, Bonn, Tokyo, and São Paulo) test datasets. Last column describes the image quality as calculated by Retinograd-AI^47^.

## Data Availability

The data that support the findings of this study are divided into two groups, published data and restricted data. Published data constitutes synthetic data derived from the Eye2Gene training dataset and are available from University College London at 10.5522/04/28604234.v1 (ref. ^48^). In combination with the code at https://github.com/Eye2Gene/Classification (ref. ^49^), this can be used to train a smaller local version of Eye2Gene. Restricted data are curated for Eye2Gene users under a licence and cannot be published, to protect patient privacy and intellectual property. Access request to Eye2Gene datasets for the purpose of collaboration can be made via a contact form on the Eye2Gene website (www.eye2gene.com).

## Code availability

The source code for model architecture training and inference is available at https://github.com/Eye2Gene/Classification (ref. ^49^). The code can also be run online via the CodeOcean capsule (https://codeocean.com/capsule/0706698/). The model weights of Eye2Gene are intellectual proprietary of UCLB so cannot be shared publicly. However, they may be shared via a licensing agreement with UCLB. A running version of the Eye2Gene web app is accessible at https://app.eye2gene.com and via the Heidelberg Appway on invitation. Access request to Eye2Gene can be made via a contact form on the Eye2Gene website (www.eye2gene.com). This is to limit risk of misuse of Eye2Gene.
