# Supplementary material for: Next-generation phenotyping of inherited retinal diseases from multimodal imaging with Eye2Gene
Source: Nat Mach Intell. 2025 Jun 18;7(6):967–78. doi: 10.1038/s42256-025-01040-8 (PMC12185311; doi:10.1038/s42256-025-01040-8)
Supplement: Supplementary file 1 — Supplementary Figs. 1–23 and Tables 1–7. [file 42256_2025_1040_MOESM1_ESM.pdf]

nature machine intelligence

Supplementary information https://doi.org/10.1038/s42256-025-01040-8

Next-generation phenotyping of inherited retinal diseases from multimodal imaging with Eye2Gene

In the format provided by the authors and unedited

Table of Content

Supplementary Figure 1: Example of the three modalities commonly used to examine the retinas of patients with inherited retinal disease. *A] Infrared (IR) fundus acquired at 30 degrees B] Fundus autofluorescence (FAF) acquired at 55 degrees C] Spectral-domain Optical Coherence Tomography (SD-OCT).* 3

Supplementary Figure 2: For each modality, five different neural networks are trained independently Each image is passed through 5 individual modality specific neural networks. *The 5 networks from each modality are ensembled by averaging to produce the 3 modality-specific components of Eye2Gene gene classification model: the FAF model, the IR model and the OCT model. The Eye2Gene model is the ensemble output of these 3 models.* 3

Supplementary Figure 3: Top-5 accuracy of Eye2Gene by ethnicity on the internal Moorfields Eye Hospital test set. *Error bars denote 95% confidence interval. Due to low patient numbers, patients were grouped according to the high-level ethnic categories from ONS recommendations. An ANOVA test did not reveal statistically significant differences between groups (p=0.220).* 4

Supplementary Figure 4: UMAP embeddings of patients based on FAF imaging, with 10 family groups highlighted. *Grey points represent patients, while other symbols represents patients from 10 different family groups numbered from 1 to 10. Patients within the same family group are usually found close to each other within the embedding space.* 5

Supplementary Figure 5: Resulting dendrogram from applying hierarchical clustering on the Eye2Gene embeddings using Euclidean distance between gene centroids with Ward linkage for a selection of 69 genes, including a number unseen by Eye2Gene. *The dendrogram recapitulates known phenotypic groupings. For example, achromatopsia genes (CNGA3 and CNGB3) marked in red, Stargardt phenocopy genes (ABCA4, PRPH2 and PROM1) marked in blue, Retinitis Pigmentosa genes (USH2A and RPGR) marked in green, Leber Congenital Amaurosis genes (RPE65 and RDH12) marked in yellow, and ocular coloboma genes (LRP2 and PAX6) marked in purple.* 6

Supplementary Figure 6: UMAP embeddings of patients based on FAF imaging, for all 170 different gene diagnoses for which patients with 55 degree FAF images were available*. Relevant patients are marked in red while other patients are marked in gray. Genes in the 63 genes classified by Eye2Gene are denoted with an asterisk.* 7

Supplementary Table 1: Results from previous studies and this study that have applied deep-learning to inherited retinal disease classification from retinal scans *(OCT=Optical Coherence Tomography and FAF=Fundus Auto Fluorescence)* 8

Supplementary Figure 7: Attention maps generated for a set of example FAF images by taking the attention weights of the initial self-attention layer of Eye2Gene (averaged across attention heads). *The attention maps show the model attends to anatomical and pathological features such as the characteristic hyper-fluorescent ring in rod-cone dystrophy.* 9

Supplementary Figure 8: Example cases from the Moorfields Eye Hospital test set with example images from the three closest matches from the internal Moorfields Eye Hospital test set according to cosine similarity of Eye2Gene autofluorescence embeddings (aggregated by patient). *This approach to interpretability is inspired by prototype-based methods[6].* 10

Supplementary Table 2: Approaches to conformal set construction and calibration. 11

Supplementary Figure 9: Coverage against average set size for our test set for 3 methods of Conformal Prediction: Least Ambiguous Adaptive Prediction Sets (LAPS), Adaptive Prediction Sets (APS) and Regularized Adaptive Prediction Sets (RAPS). *Note that at a coverage value of 0.8, LAPS produces prediction sets with almost half the size of the second best-performing method (RAPS).* 11

Supplementary Figure 10: Overview of how Eye2Gene can be implemented in the inherited retinal disease clinical pathway in helping to improve diagnostic yield. 12

Supplementary Figure 11: Sensitivity of Eye2Gene per gene compared to number of images (log scale) in the training data. 13

Supplementary Figure 12: The distribution of patient ethnicities per gene in our training dataset across 36 unique genes*. Certain recessive genes such as CERKL and CYP4V2 tend to be more prevalent in South Asians. Overall ethnicity is often not stated.* 13

Supplementary Table 2: Ethnicity coding of Moorfields Eye Hospital Inherited Retinal Disease cohort in Eye2Gene dataset. 44% of patients do not disclose their ethnicity or have an unknown ethnicity. *A mix of different ethnic backgrounds is represented however certain ethnicities are under-represented compared to the global population. Since IRD gene distributions vary across different populations – mostly due to founder effects [7,8] – which is likely to impact the generalizability of Eye2Gene. Hence more data is needed to ensure Eye2Gene is assessed across different ethnicities, as well as across different types of patient populations.* 14
Supplementary Figure 13: Geographic distribution of patients affected by Inherited Retinal Diseases seen at Moorfields Eye Hospital. 15
Supplementary Figure 14: Retinograd-AI[9] gradeability scores of retinal scans which are correctly and incorrectly classified by Eye2Gene. *Gradability is lower in incorrectly classified scans.* 16
Supplementary Figure 15: Number of patients per gene class for all 189 genes in our initial dataset. Genes in red had fewer than 10 patients and so were excluded from experiments. *The remaining genes in blue amount to 63 genes in total that were used to train Eye2Gene.* 17
Supplementary Figure 16: Quality control filtering process on the entire Moorfields Eye Hospital inherited retinal disease dataset. *As a result of the filtering, 25,233 FAF, 31,357 IR and 124,975 OCT scans remain in 63 most common genes across 3,652 patients.* 18
Supplementary Table 4: Genotypes and associated phenotypic presentation of 2103 patients. *Citations provided to natural history studies in which the phenotypes of some of these patients are described.* 19
Supplementary Table 5: Phenotypic presentation of 2103 patients* 20
Supplementary Figure 17: Distributions of visual acuity (in LogMar) with respect to 36 genes sorted by the median of the visual acuity distribution per gene. *Low vision is defined as a best-corrected visual acuity worse than 0.5 LogMAR but equal or better than 1.3 LogMAR in the better eye. Blindness is defined as a best-corrected visual acuity worse than 1.3 LogMAR. Also represented are Logmar of 1.98 (Counting Fingers), 2.28 (Hand Movement) and 2.7 (Light Perception).* 21
Supplementary Table 6: *Detailed overview of the test dataset for Eye2Gene. P=patients; FAF=fundus autofluorescence; IR=infrared; OCT=optical coherence tomography;* 22
Supplementary Figure 18: Example network training loss curves. *100 epochs was found to be sufficient for training to converge for a wide variety of hyper-parameter settings in preliminary investigations.* 24
Supplementary Figure 19: Data augmentation transformations applied to the training set. *Data augmentation techniques were applied to the training data which were felt to be realistic transformations of the data. These included random brightness adjustment, horizontal flip for all three modalities and rotations by up to 15 degrees.* 24
Supplementary Figure 20: Top-5 accuracy of Eye2Gene on the all test data subsamling k images per patient was randomly subsampled (mean over 10 trials) for different values of k (k=1,2,3,5,10,20,30,50,100). 25
Supplementary Table 7: Additional metrics on full validation data. 25
Supplementary Figure 21: Per-gene Receiver Operating Characteristic (ROC) curves of Eye2Gene on the Moorfields Eye Hospital internal test data for the three different imaging modalities across the 63 different predicted genes. *Percentages next to gene name denote the percentage of total patients corresponding to the given gene.* 26
Supplementary Figure 22: UMAP-projected embeddings of FAF images from external sites overlaid over the internal MEH embeddings. *Points were projected using the UMAP model derived from the Moorfields data using the python umap-learn package.* 27
Supplementary Figure 23: Autofluorescence images and corresponding attention heatmaps produced by Eye2Gene (a) *RS1 retinopathy is typically characterized by macular schisis which appears as reduced autofluorescence due to structural disruption of the retinal layers and is highlighted in the heatmap by the orange and red pixels.* ***(b)*** *BEST1 retinopathy is characterized by vitelliform areas of increased autofluorescence and are highlighted on the heatmap by the orange and red pixels* ***(c)*** *PDE6C retinopathy characterized by reduced autofluorescence at the fovea and a bright ring of autofluorescence due to severe cone dysfunction/loss, which are highlighted by the red and orange pixels on the corresponding heatmap.* ***(d)*** *PRPH2 retinopathy is characterized by dispersed peripheral focal areas of hypo and a ring-like hyper-autofluorescence which are highlighted by the red and orange pixels in the corresponding heatmap.* 27

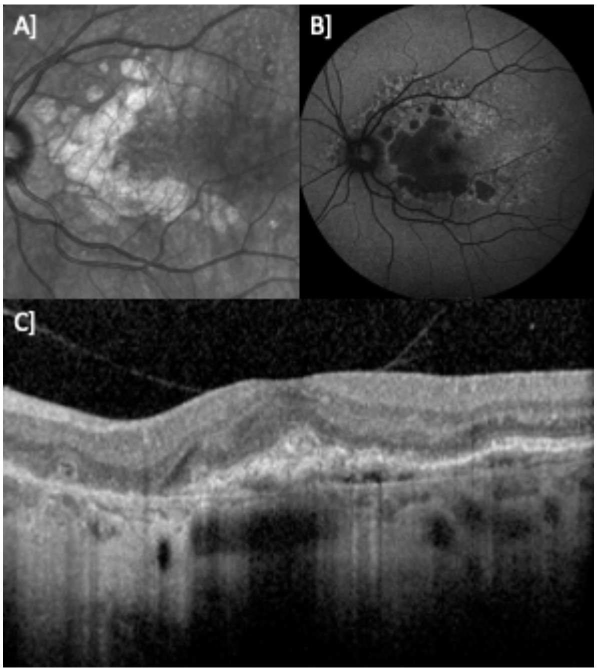

Supplementary Figure 1: Example of the three modalities commonly used to examine the retinas of patients with inherited retinal disease. A] Infrared (IR) fundus acquired at 30 degrees B] Fundus autofluorescence (FAF) acquired at 55 degrees C] Spectral-domain Optical Coherence Tomography (SD-OCT).

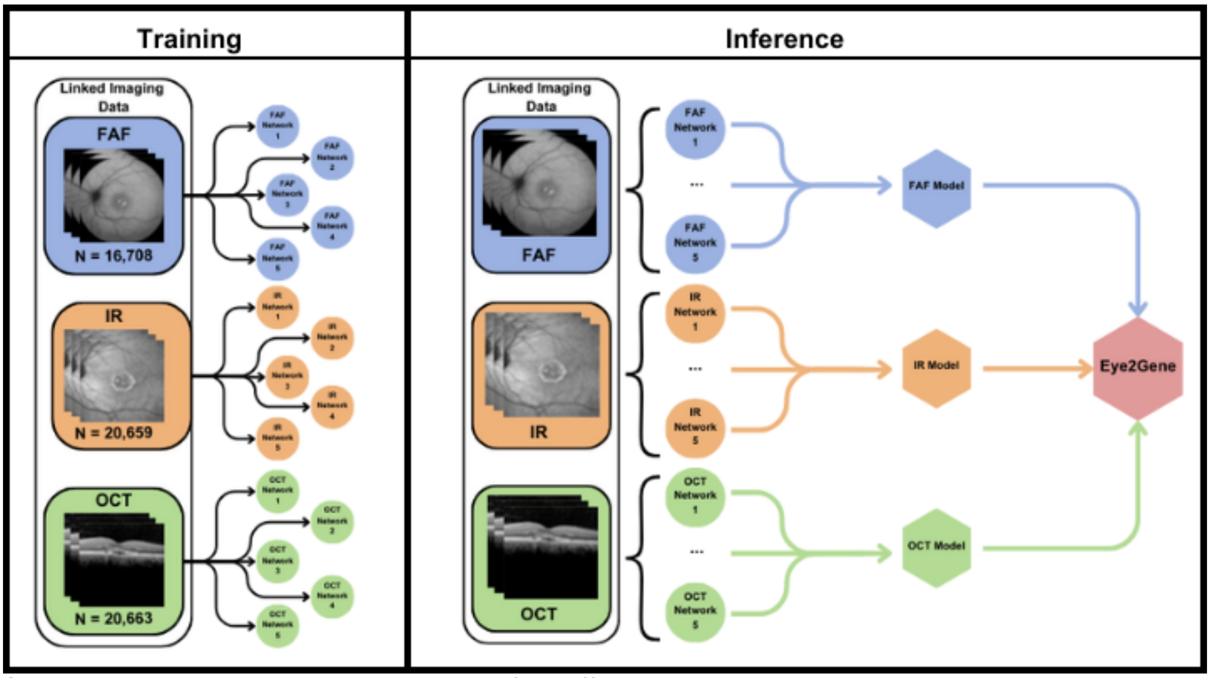

Supplementary Figure 2: For each modality, five different neural networks are trained independently Each image is passed through 5 individual modality specific neural networks. The 5 networks from each modality are ensembled by averaging to produce the 3 modality-specific components of Eye2Gene gene classification model: the FAF model, the IR model and the OCT model. The Eye2Gene model is the ensemble output of these 3 models.

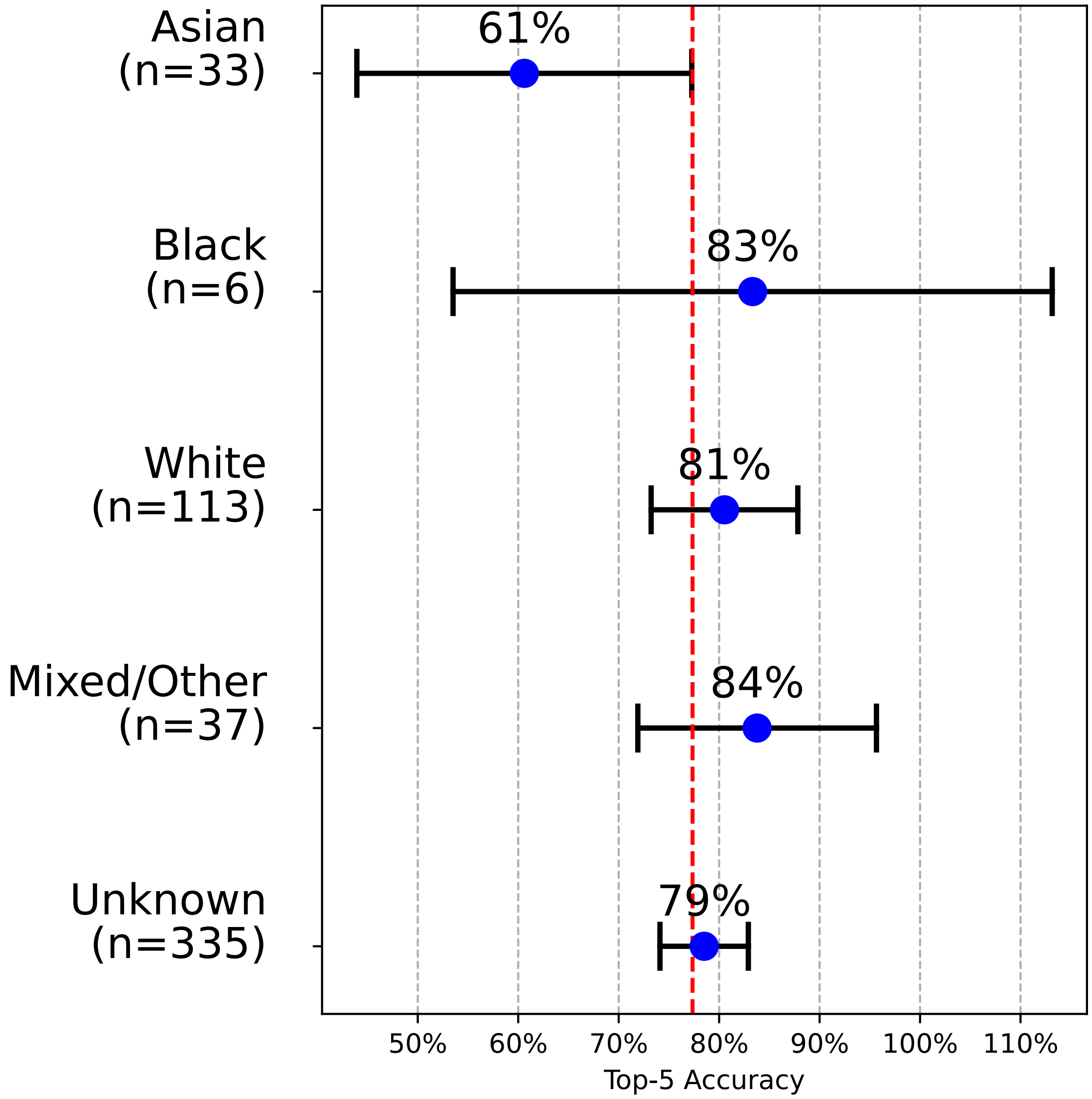

Supplementary Figure 3: Top-5 accuracy of Eye2Gene by ethnicity on the internal Moorfields Eye Hospital test set. Error bars denote 95% confidence interval. Due to low patient numbers, patients were grouped according to the high-level ethnic categories from ONS recommendations. An ANOVA test did not reveal statistically significant differences between groups (p=0.220).

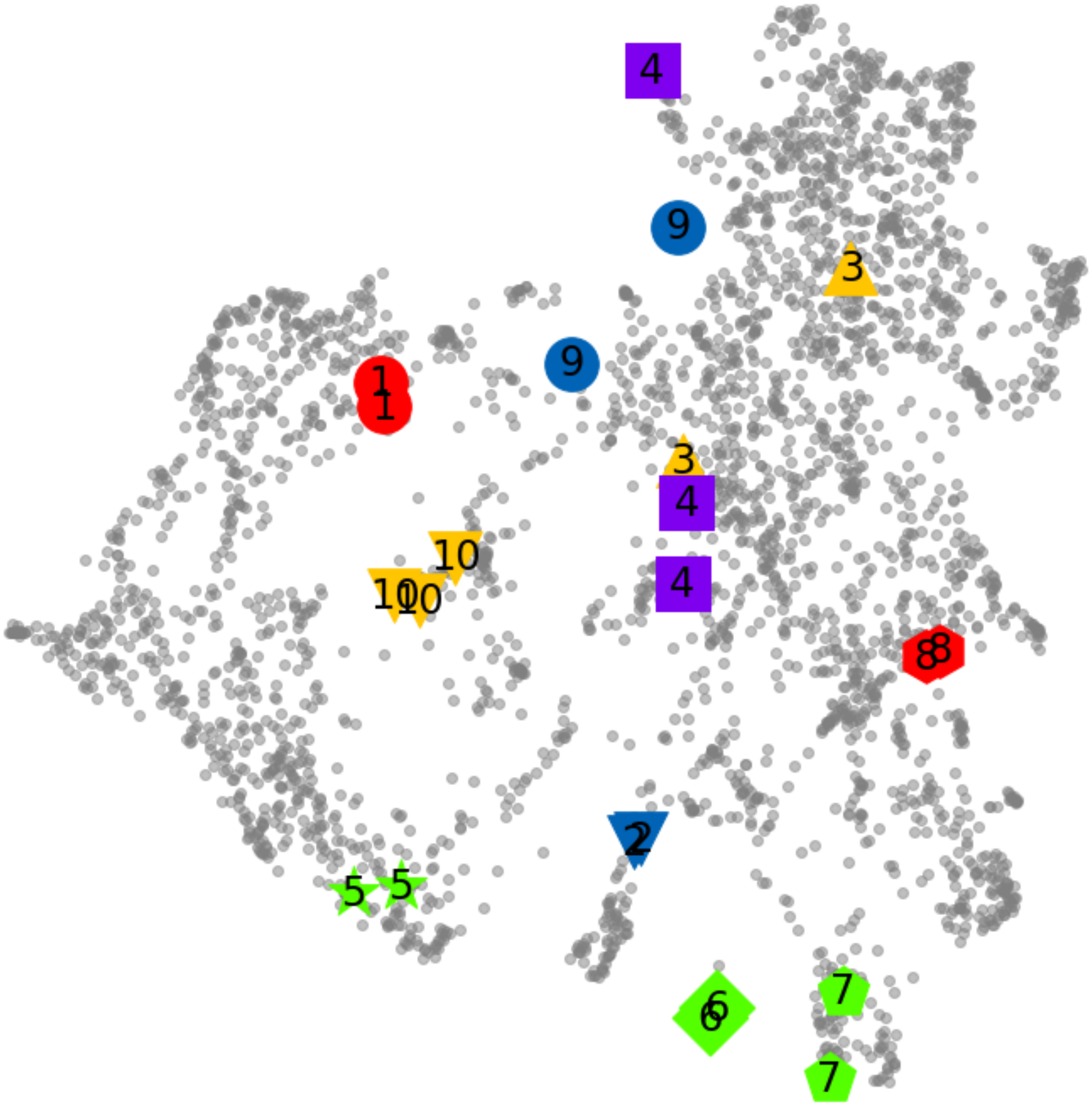

Supplementary Figure 4: UMAP embeddings of patients based on FAF imaging, with 10 family groups highlighted. Grey points represent patients, while other symbols represents patients from 10 different family groups numbered from 1 to 10. Patients within the same family group are usually found close to each other within the embedding space.

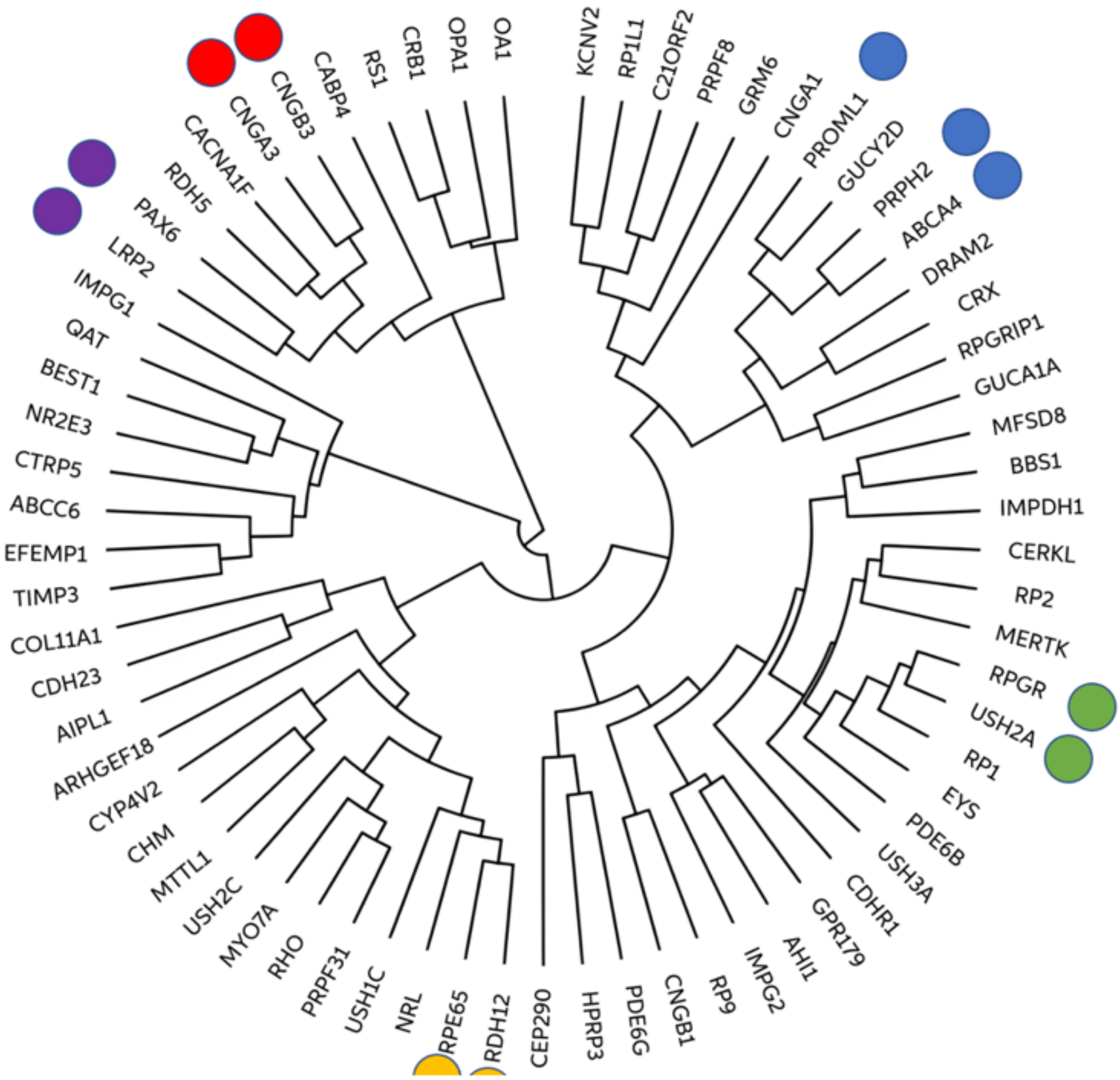

Supplementary Figure 5: Resulting dendrogram from applying hierarchical clustering on the Eye2Gene embeddings using Euclidean distance between gene centroids with Ward linkage for a selection of 69 genes, including a number unseen by Eye2Gene. The dendrogram recapitulates known phenotypic groupings. For example, achromatopsia genes (*CNGA3* and *CNGB3*) marked in red, Stargardt phenocopy genes (*ABCA4*, *PRPH2* and *PROM1*) marked in blue, Retinitis Pigmentosa genes (*USH2A* and *RPGR*) marked in green, Leber Congenital Amaurosis genes (*RPE65* and *RDH12*) marked in yellow, and ocular coloboma genes (*LRP2* and *PAX6*) marked in purple.

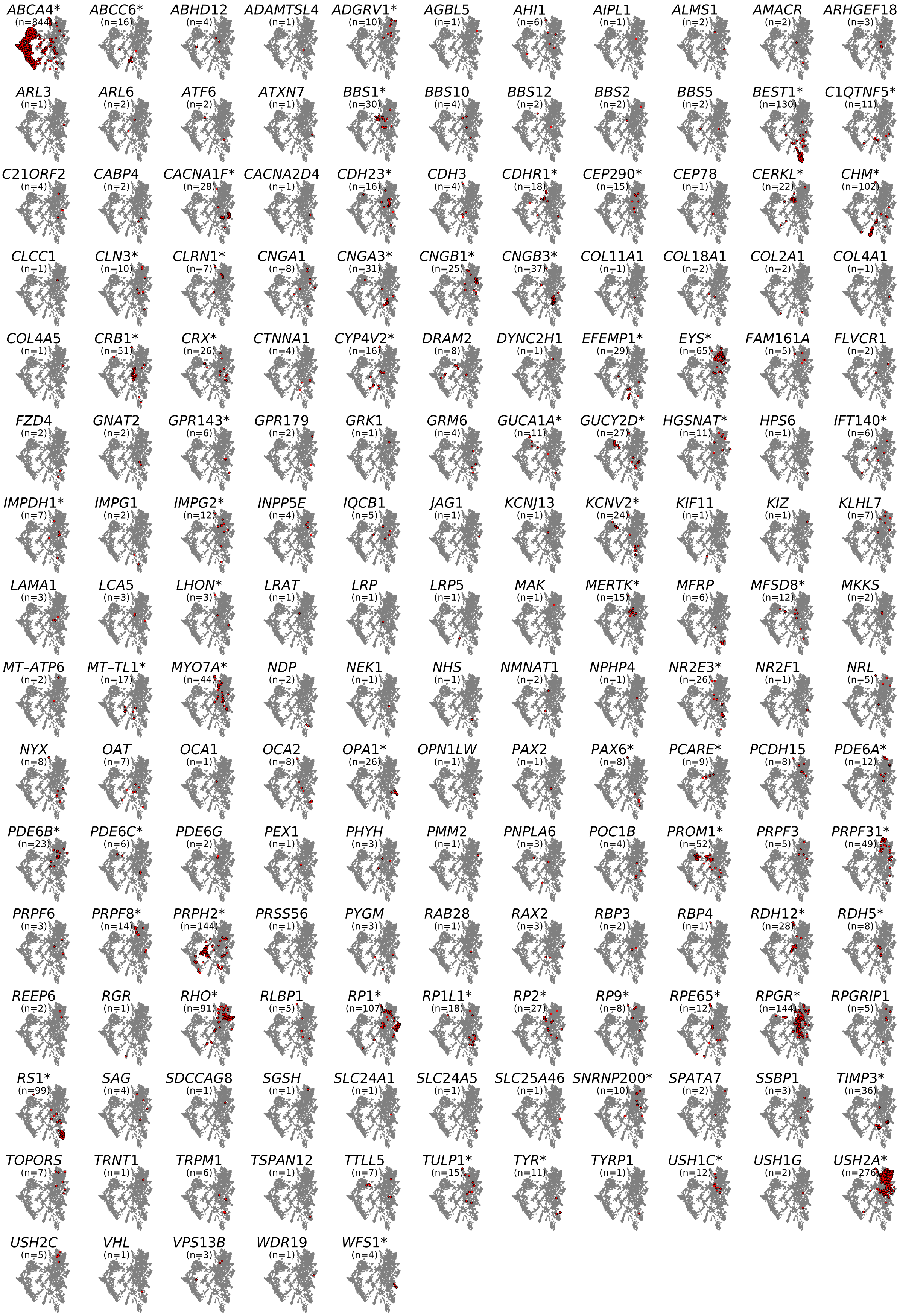

Supplementary Figure 6: UMAP embeddings of patients based on FAF imaging, for all 170 different gene diagnoses for which patients with 55 degree FAF images were available. Relevant patients are marked in red while other patients are marked in gray. Genes in the 63 genes classified by Eye2Gene are denoted with an asterisk.

Supplementary Table 1: Results from previous studies and this study that have applied deep-learning to inherited retinal disease classification from retinal scans (OCT=Optical Coherence Tomography and FAF=Fundus Auto Fluorescence)

Previous studies:

Publication	Data Prep	Network	Image Type	Classes	Patient Count	Image Count	Sens (%)	Spec (%)	Acc (%)
(Fujinami-Yokokawa et al., 2019)[1]	Centred cropped: 750×500	InceptionV3	OCT	*ABCA4*<br>*RP1L1*<br>*EYS*<br>Normal	10<br>20<br>28<br>17	19<br>37<br>57<br>65	81.7<br>78.2<br>89.7<br>93.4	100<br>95<br>95.3<br>96	89.3
(Shah et al., 2020)[2]	Centred cropped: 256×256	VGG19	OCT	Stargardt<br>Normal	60<br>33	647<br>102	99.8<br>98	98<br>99.8	99.6
(Miere et al., 2020)[3]	Centred cropped: 768×768	ResNet 101	FAF	Stargardt<br>Retinitis Pigmentosa<br>Best Disease<br>Normal	N/S<br>N/S<br>N/S<br>N/S	125<br>160<br>125<br>73	96<br>100<br>92<br>86	100<br>97<br>97<br>99	94.6
(Fujinami-Yokokawa et al., 2021)[4]	Centred cropped: 500x500	InceptionV3	FAF	*ABCA4*<br>*EYS*<br>*RP1L1*<br>Normal	19<br>18<br>22<br>23	37<br>35<br>43<br>43	97.5<br>70.8<br>65<br>93	95<br>99.2<br>96.5<br>84.2	81.5
(Miere et al., 2021)[5]	N/S	ResNet50V2	FAF	*ABCA4*<br>*PRPH2*	40<br>9	304<br>66	96.7<br>50	50<br>96.7	88

Our results (predictions from Eye2Gene were restricted to the specified genes) **:**

Data Prep	Network	Image Type	Classes	Patient Count		Image Count		Sens (%)	Spec (%)	Acc (%)
				Train	Test	Train	Test			
768x768	Eye2Gene FAF Only	FAF	*ABCA4*<br>*BEST1*<br>*USH2A*	588<br>93<br>188	331<br>65<br>81	4709<br>898<br>1465	1383<br>225<br>366	97.6<br>86.2<br>95.1	93.8<br>99.0<br>98.0	95.6
768x768 (FAF/IR) 496x512 (OCT)	Eye2Gene	FAF / IR / OCT	*ABCA4*<br>*BEST1*<br>*USH2A*	666<br>106<br>238	373<br>83<br>87	33763<br>4776<br>11892	9646<br>1472<br>2861	97.3<br>91.6<br>96.6	95.3<br>98.5<br>98.9	96.3
768x768	Eye2Gene FAF Only	FAF	*ABCA4*<br>*EYS*<br>*RP1L1*	588<br>48<br>10	331<br>11<br>5	4709<br>317<br>97	1383<br>59<br>29	98.2<br>45.5<br>80	62.5<br>99.1<br>98.8	96.3
768x768 (FAF/IR) 496x512 (OCT)	Eye2Gene	FAF / IR / OCT	*ABCA4*<br>*EYS*<br>*RP1L1*	666<br>56<br>11	373<br>22<br>5	33763<br>2111<br>652	9646<br>334<br>141	98.7<br>45.5<br>80	59.3<br>98.9<br>99.2	95.5
768x768	Eye2Gene FAF Only	FAF	*ABCA4*<br>*PRPH2*	588<br>97	331<br>73	4709<br>822	1383<br>236	97.6<br>60.3	60.3<br>97.6	90.8
768x768 (FAF/IR) 496x512 (OCT)	Eye2Gene	FAF / IR / OCT	*ABCA4*<br>*PRPH2*<br>*PROM1*<br>*CACNA1F*	666<br>112<br>36<br>25	373<br>95<br>24<br>6	33763<br>4837<br>1303<br>747	9646<br>1575<br>443<br>125	97.1<br>58.9<br>4.2<br>100	55.2<br>96.8<br>99.4<br>99.8	85.3

Italic is used for gene names.

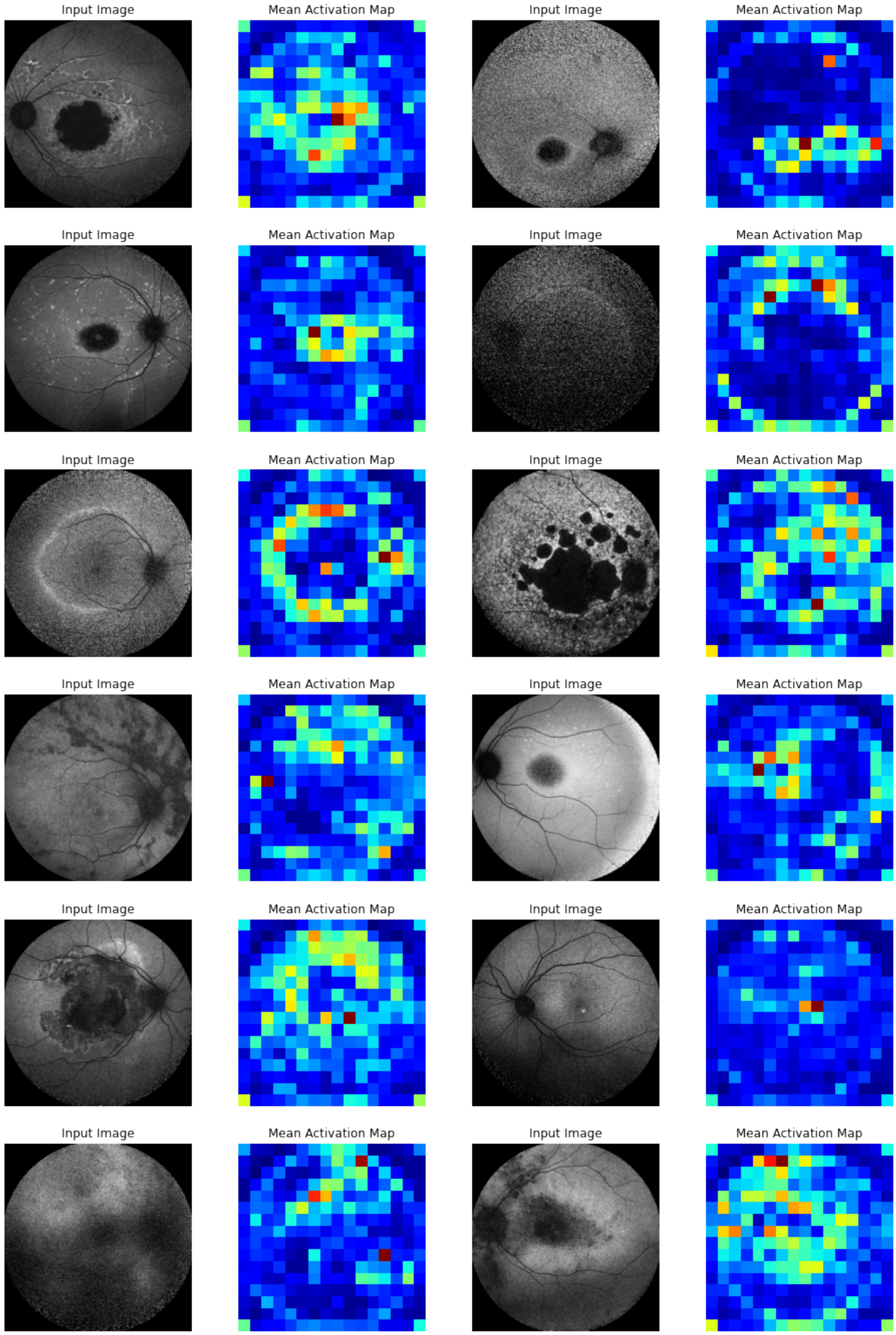

Supplementary Figure 7: Attention maps generated for a set of example FAF images by taking the attention weights of the initial self-attention layer of Eye2Gene (averaged across attention heads). The attention maps show the model attends to anatomical and pathological features such as the characteristic hyper-fluorescent ring in rod-cone dystrophy.

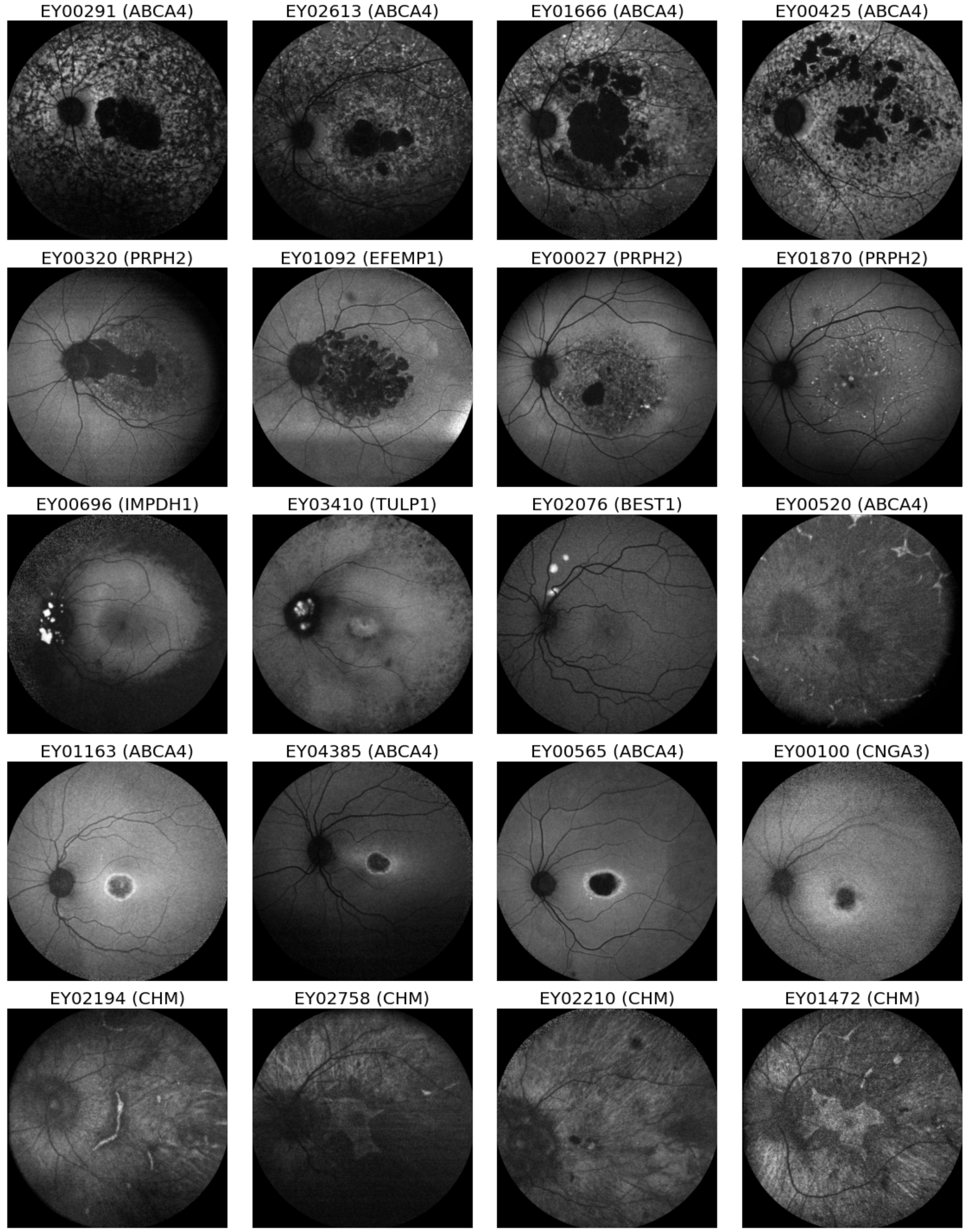

Supplementary Figure 8: Example cases from the Moorfields Eye Hospital test set with example images from the three closest matches from the internal Moorfields Eye Hospital test set according to cosine similarity of Eye2Gene autofluorescence embeddings (aggregated by patient). This approach to interpretability is inspired by prototype-based methods[6].

Supplementary Table 2: Approaches to conformal set construction and calibration.

Method	Description	Advantages
Least Ambiguous Prediction Sets (LAPS)	Calibrate using percentile of predicted likelihood of true class on calibration set.	Calibrated to achieve desired coverage threshold.
Adaptive Prediction Sets (APS)	Calibrate using percentile of calculated threshold of true class on calibration set.	Reduce over/under covering of common/rare classes
Regularized APS (RAPS)	Addition of regularization parameter penalizing larger prediction sets	Controls prediction set size for more Ambiguous cases

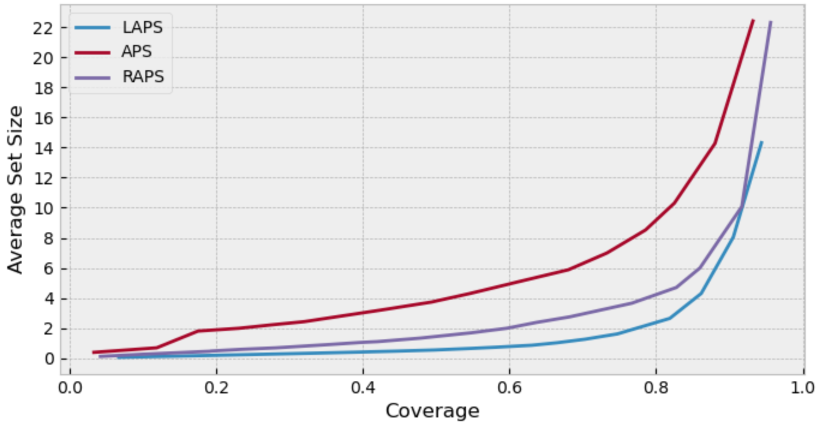

Supplementary Figure 9: Coverage against average set size for our test set for 3 methods of Conformal Prediction: Least Ambiguous Adaptive Prediction Sets (LAPS), Adaptive Prediction Sets (APS) and Regularized Adaptive Prediction Sets (RAPS). Note that at a coverage value of 0.8, LAPS produces prediction sets with almost half the size of the second best-performing method (RAPS).

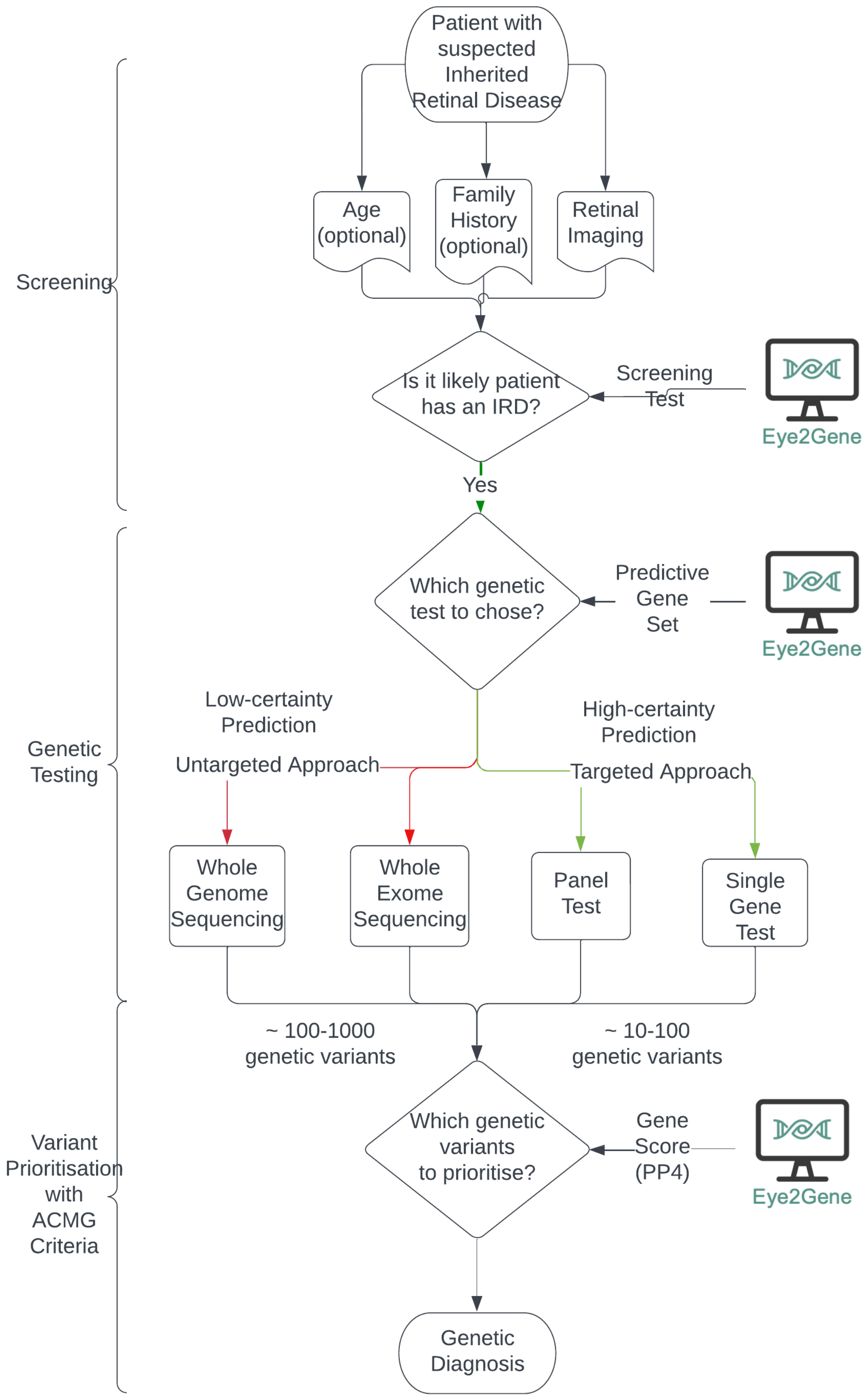

Supplementary Figure 10: Overview of how Eye2Gene can be implemented in the inherited retinal disease clinical pathway in helping to improve diagnostic yield.

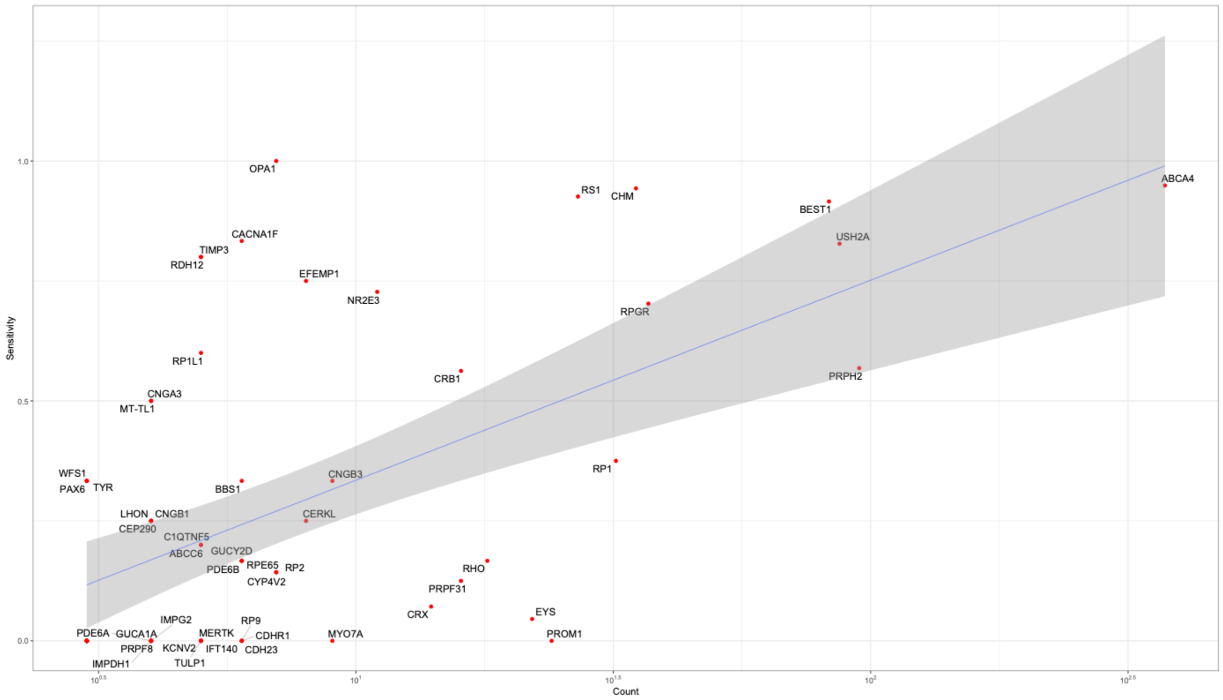

Supplementary Figure 11: Sensitivity of Eye2Gene per gene compared to number of images (log scale) in the training data.

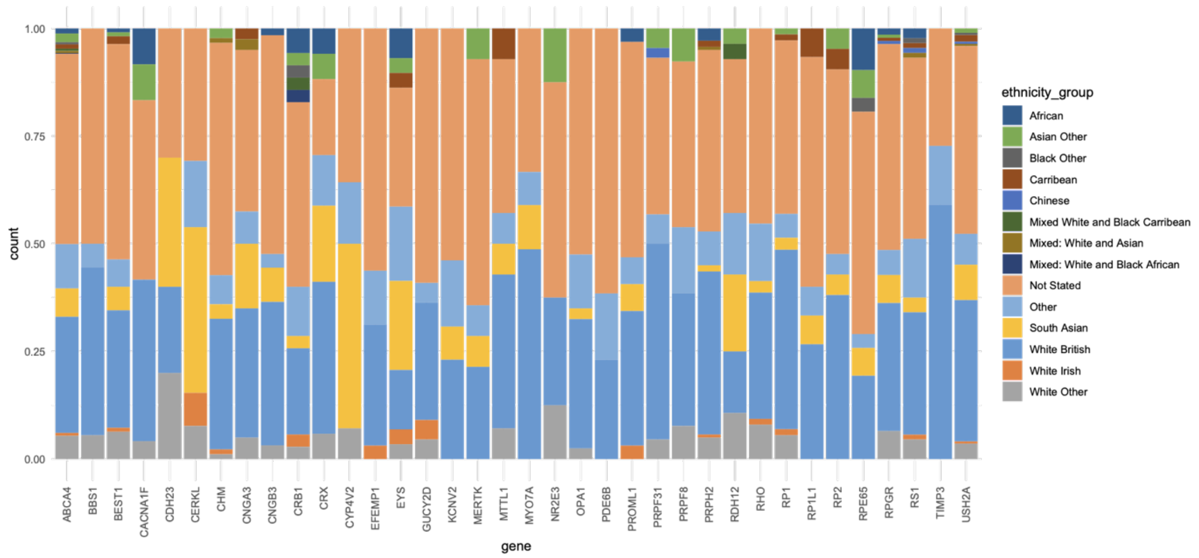

Supplementary Figure 12: The distribution of patient ethnicities per gene in our training dataset across 36 unique genes. Certain recessive genes such as *CERKL* and *CYP4V2* tend to be more prevalent in South Asians. Overall ethnicity is often not stated.

Supplementary Table 3: Ethnicity coding of Moorfields Eye Hospital Inherited Retinal Disease cohort in Eye2Gene dataset. 44% of patients do not disclose their ethnicity or have an unknown ethnicity. A mix of different ethnic backgrounds is represented however certain ethnicities are under-represented compared to the global population. Since IRD gene distributions vary across different populations – mostly due to founder effects [7,8] – which is likely to impact the generalizability of Eye2Gene. Hence more data is needed to ensure Eye2Gene is assessed across different ethnicities, as well as across different types of patient populations.

Ethnicity	Percentage
Not stated	37.00
Unknown	7.51
British	30.09
Any other ethnic group	8.20
Any other White background	4.65
Indian	3.41
Pakistani	1.84
Any other Asian background	1.57
African	1.29
Bangladeshi	1.01
Caribbean	0.97
Irish	0.78
Any other mixed background	0.55
White and Asian	0.37
Any other Black background	0.32
Chinese	0.18
White and Black Caribbean	0.14
White and Black African	0.09

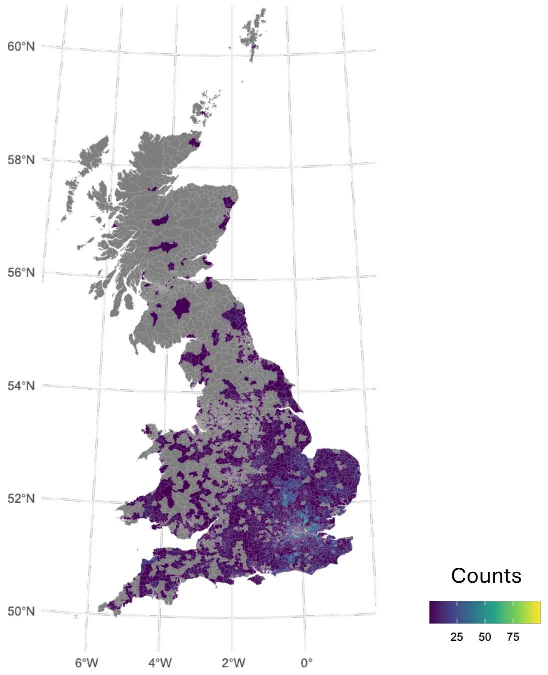

Supplementary Figure 13: Geographic distribution of patients affected by Inherited Retinal Diseases seen at Moorfields Eye Hospital.

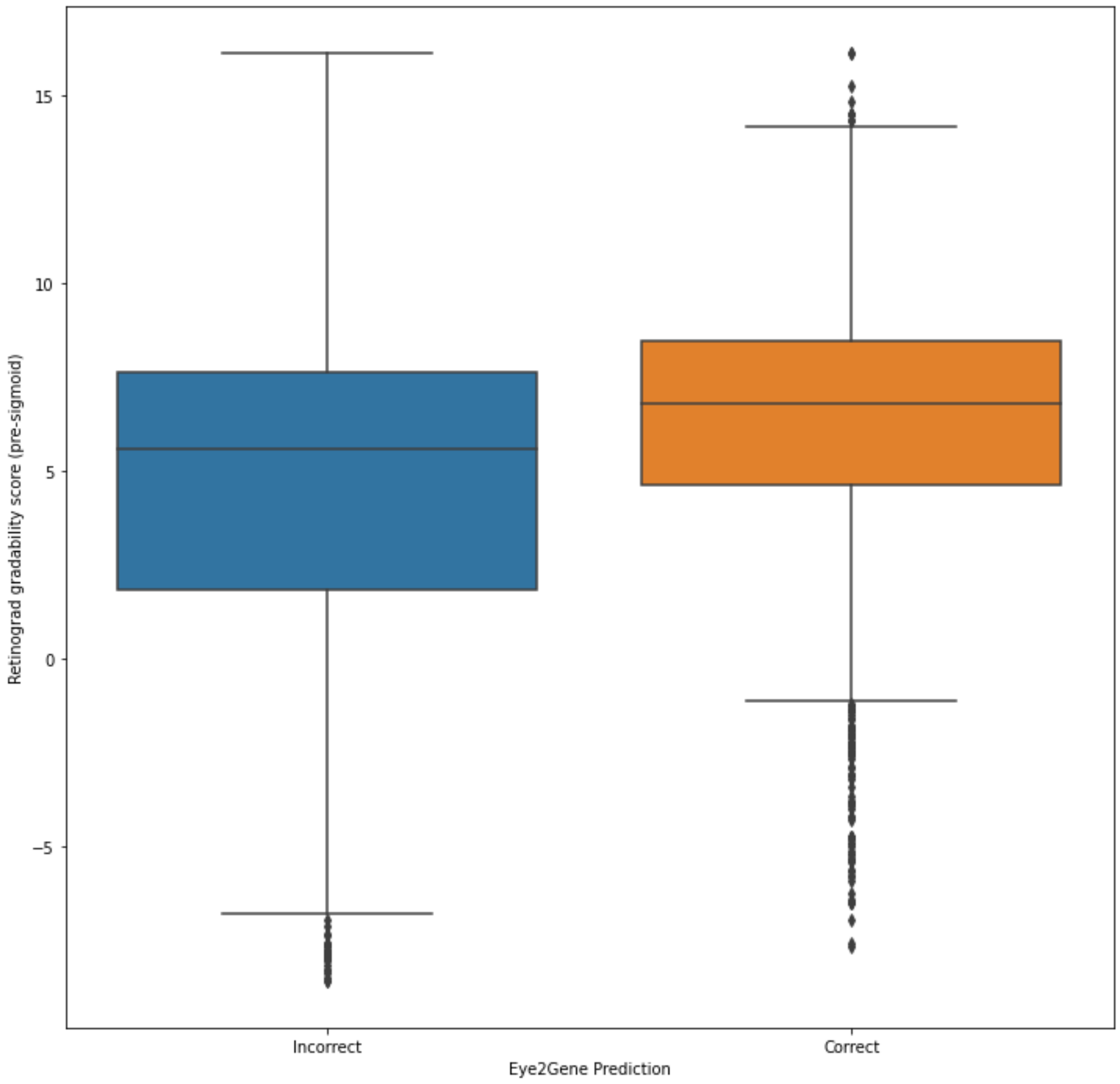

Supplementary Figure 14: Retinograd-AI[9] gradeability scores of retinal scans which are correctly and incorrectly classified by Eye2Gene. Gradability is significantly lower in incorrectly classified scans ($p<0.001$).

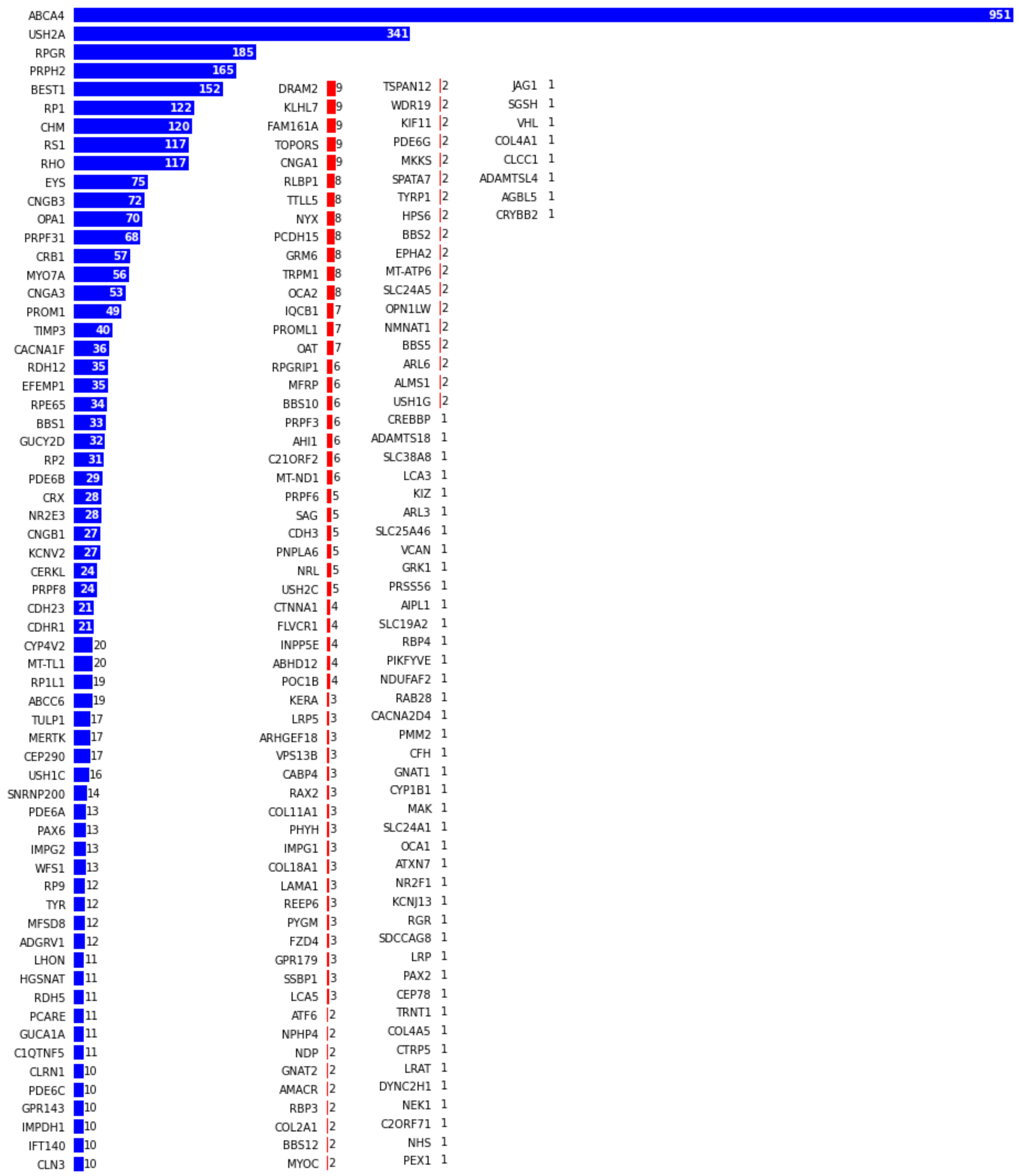

Supplementary Figure 15: Number of patients per gene class for all 189 genes in our initial dataset. Genes in red had fewer than 10 patients and so were excluded from experiments. The remaining genes in blue amount to 63 genes in total that were used to train Eye2Gene.

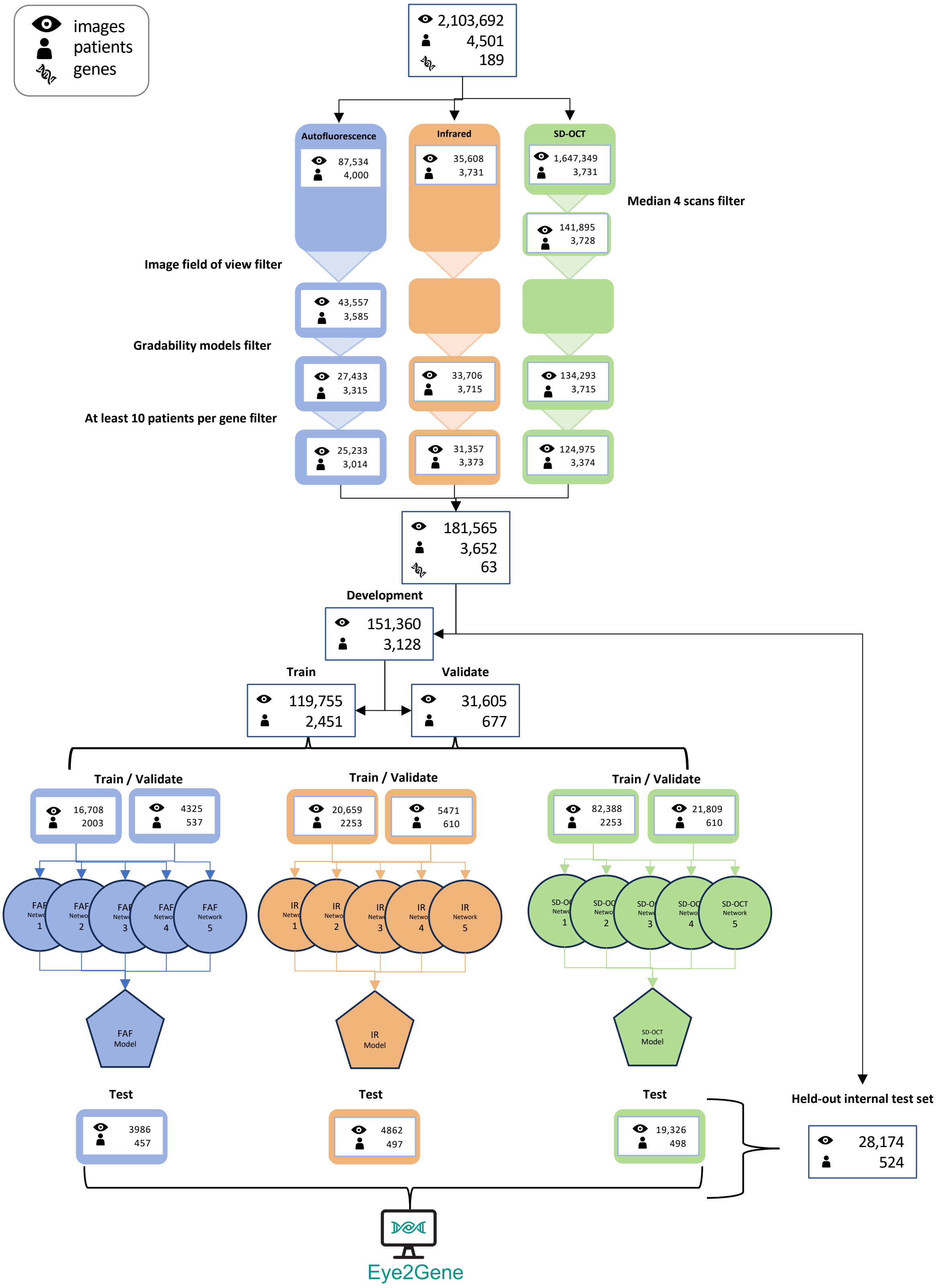

Supplementary Figure 16: Quality control filtering process on the entire Moorfields Eye Hospital inherited retinal disease dataset. As a result of the filtering, 25,233 FAF, 31,357 IR and 124,975 OCT scans remain in 63 most common genes across 3,652 patients.

Supplementary Table 4: Genotypes and associated phenotypic presentation of 2103 patients. Citations provided to natural history studies in which the phenotypes of some of these patients are described.

Genotype	Phenotype	n=	Percentage (%)
ABCA4[10,11]	Stargardt Disease	575	27.3%
BBS1	Retinitis Pigmentosa	18	0.9%
BEST1[12]	Best Disease	106	5.0%
CACNA1F	Congenital Stationary night Blindness	23	1.1%
CDH23	Retinitis Pigmentosa	10	0.5%
CERKL	Cone-Rod Dystrophy	13	0.6%
CHM	Choroideremia	87	4.1%
CNGA3[13]	Achromatopsia	40	1.9%
CNGB3	Achromatopsia	63	3.0%
CRB1[14]	Leber Congenital Amaurosis/Early-Onset Severe Retinal Dystrophy	14	0.7%
	Retinitis Pigmentosa	20	1.0%
CRX	Leber Congenital Amaurosis/Early-Onset Severe Retinal Dystrophy	17	0.8%
CYP4V2	Bietti Crystalline Corneoretinal Dystrophy	13	0.6%
EFEMP1	Autosomal Dominant Drusen	30	1.4%
EYS	Retinitis Pigmentosa	30	1.4%
GUCY2D[15,16]	Cone-Rod Dystrophy	12	0.6%
	Leber Congenital Amaurosis/Early-Onset Severe Retinal Dystrophy	10	0.5%
KCNV2[17]	Cone-Rod Dystrophy	13	0.6%
MERTK	Leber Congenital Amaurosis/Early-Onset Severe Retinal Dystrophy	7	0.3%
	Retinitis Pigmentosa	7	0.3%
MTTL1	Mitochondrial Retinopathy	14	0.7%
MYO7A	Retinitis Pigmentosa	37	1.8%
NR2E3	Enhanced S-Cone Syndrome	15	0.7%
OPA1	Autosomal Dominant Optic Atrophy	40	1.9%
PDE6B	Retinitis Pigmentosa	13	0.6%
PROM1	Retinitis Pigmentosa	30	1.4%
PRPF31	Retinitis Pigmentosa	43	2.0%
PRPF8	Retinitis Pigmentosa	11	0.5%
PRPH2	Pattern Dystrophy	133	6.3%
RDH12[18,19]	Cone-Rod Dystrophy	3	0.1%
	Leber Congenital Amaurosis/Early-Onset Severe Retinal Dystrophy	25	1.2%
RHO	Retinitis Pigmentosa	70	3.3%
RP1	Retinitis Pigmentosa	68	3.2%
RP1L1	Retinitis Pigmentosa	14	0.7%
RP2[20]	Retinitis Pigmentosa	20	1.0%
RPE65[21]	Leber Congenital Amaurosis/Early-Onset Severe Retinal Dystrophy	31	1.5%
RPGR[22,23]	Cone-Rod Dystrophy	13	0.6%
	Retinitis Pigmentosa	120	5.7%
RS1[24]	X-Linked Retinoshisis	88	4.2%
TIMP3	Sorsby Fundus Dystrophy	21	1.0%
USH2A	Retinitis Pigmentosa	186	8.8%

Italic is used for gene names.

Supplementary Table 5: Phenotypic presentation of 2103 patients*

Phenotype	n=	Percentage (%)
**Macular Dystrophies**	**953**	**45.3%**
Stargardt Disease**	575	27.3%
Best Disease	106	5.0%
X-Linked Retinoschisis	88	4.2%
Pattern Dystrophy	133	6.3%
Sorsby Fundus Dystrophy	21	1.0%
Autosomal Dominant Drusen	30	1.4%
**Cone and Cone-Rod Dystrophies****	**54**	**2.6%**
**Cone Dysfunction Syndromes**	**103**	**4.9%**
Achromatopsia	103	4.9%
**Leber Congenital Amaurosis/Early-Onset Severe Retinal Dystrophy**	**104**	**4.9%**
**Rod-Cone Dystrophy**	**829**	**39.4%**
Retinitis Pigmentosa	801	38.1%
Enhanced S-Cone Syndrome	15	0.7%
Bietti Crystalline Corneoretinal Dystrophy	13	0.6%
**Rod Dysfunction Syndromes**	**23**	**1.1%**
Congenital Stationary night Blindness	23	1.1%
**Chorioretinal Dystrophy**	**87**	**4.1%**
Choroideremia	87	4.1%
**Autosomal Dominant Optic Atrophy**	**40**	**1.9%**
**Mitochondrial Retinopathy**	**14**	**0.7%**

* Most of the patients were previously published in genotype specific natural history studies with in-depth phenotyping. Please refer to relevant literature by the authors for more details.
***ABCA4*-associated cases are classified under Stargardt Disease

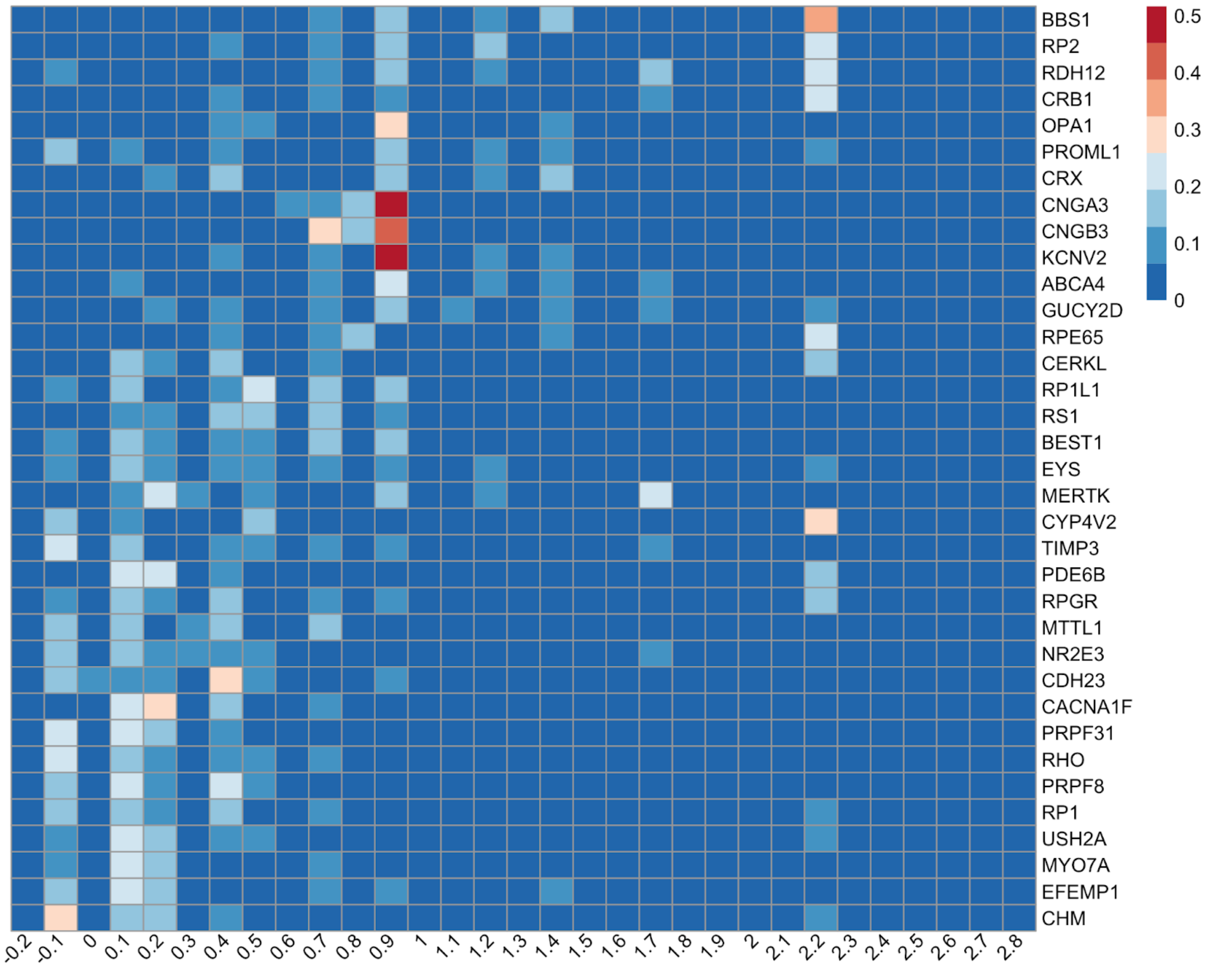

Supplementary Figure 17: Distributions of visual acuity (in LogMar) with respect to 36 genes sorted by the median of the visual acuity distribution per gene. Low vision is defined as a best-corrected visual acuity worse than 0.5 LogMAR but equal or better than 1.3 LogMAR in the better eye. Blindness is defined as a best-corrected visual acuity worse than 1.3 LogMAR. Also represented are Logmar of 1.98 (Counting Fingers), 2.28 (Hand Movement) and 2.7 (Light Perception).

Supplementary Table 6: Detailed overview of the test dataset for Eye2Gene. P=patients; FAF=fundus autofluorescence; IR=infrared; OCT=optical coherence tomography;

	Oxford				Tokyo				Sao Paulo				Liverpool				Bonn				Moorfields			
**Gene**	**P**	**FAF**	**IR**	**OCT**	**P**	**FAF**	**IR**	**OCT**	**P**	**FAF**	**IR**	**OCT**	**P**	**FAF**	**IR**	**OCT**	**P**	**FAF**	**IR**	**OCT**	**P**	**FAF**	**IR**	**OCT**
ABCA4	176	308	347	12451	8	49	43	43	18	16	49	615	42	82	82	1678	39	78	390	390	114	947	1235	4929
ABCC6	0	0	0	0	0	0	0	0	0	0	0	0	0	0	0	0	0	0	0	0	5	83	136	544
ADGRV1	0	0	0	0	0	0	0	0	0	0	0	0	0	0	0	0	0	0	0	0	3	15	31	124
BBS1	1	2	2	86	0	0	0	0	0	0	0	0	1	2	2	38	0	0	0	0	4	35	19	70
BEST1	47	65	91	3259	1	2	2	2	1	25	8	163	3	6	6	114	16	32	160	160	16	128	157	625
C1QTNF5	1	2	2	74	0	0	0	0	0	0	0	0	0	0	0	0	0	0	0	0	5	20	32	128
CACNA1F	1	0	2	74	0	0	0	0	0	0	0	0	1	2	2	38	0	0	0	0	4	13	18	72
CDH23	1	2	2	58	0	0	0	0	0	0	0	0	1	2	2	38	0	0	0	0	4	33	33	132
CDHR1	1	2	2	38	0	0	0	0	0	0	0	0	0	0	0	0	0	0	0	0	6	32	32	128
CEP290	2	4	4	116	0	0	0	0	0	0	0	0	1	2	2	38	0	0	0	0	3	62	14	56
CERKL	2	4	4	172	0	0	0	0	1	0	8	54	1	2	2	56	0	0	0	0	7	77	50	197
CHM	11	19	23	862	2	0	4	4	7	8	30	22	0	0	0	0	0	0	0	0	21	201	153	612
CLN3	0	0	0	0	0	0	0	0	0	0	0	0	0	0	0	0	0	0	0	0	3	28	33	132
CLRN1	0	0	0	0	0	0	0	0	0	0	0	0	0	0	0	0	0	0	0	0	3	4	26	101
CNGA3	0	0	0	0	0	0	0	0	0	0	0	0	0	0	0	0	0	0	0	0	4	17	17	62
CNGB1	0	0	0	0	1	0	2	2	0	0	0	0	1	2	2	38	0	0	0	0	4	16	26	104
CNGB3	6	12	11	404	0	0	0	0	1	0	2	38	0	0	0	0	1	2	0	10	7	40	129	480
CRB1	2	4	4	86	1	0	2	2	1	0	5	15	3	6	6	114	4	8	40	40	9	116	82	322
CRX	7	13	14	561	1	2	2	2	0	0	0	0	3	6	6	162	0	0	0	0	6	97	52	208
CYP4V2	0	0	0	0	2	0	4	4	0	0	0	0	0	0	0	0	0	0	0	0	6	12	23	92
EFEMP1	4	6	9	333	0	0	0	0	0	0	0	0	1	2	2	38	0	0	0	0	6	57	45	180
EYS	4	8	8	296	10	27	40	289	1	58	15	111	2	4	4	76	3	6	30	30	6	47	42	168
GPR143	0	0	0	0	0	0	0	0	0	0	0	0	0	0	0	0	0	0	0	0	3	6	10	34
GUCA1A	0	0	0	0	1	4	2	2	0	0	0	0	1	2	2	38	0	0	0	0	3	39	22	88
GUCY2D	1	2	2	74	1	2	2	2	0	0	0	0	3	6	6	114	0	0	0	0	3	6	14	56
HGSNAT	0	0	0	0	0	0	0	0	0	0	0	0	0	0	0	0	0	0	0	0	3	14	58	232
IFT140	0	0	0	0	0	0	0	0	0	0	0	0	1	2	2	38	0	0	0	0	4	30	35	137
IMPDH1	0	0	0	0	0	0	0	0	0	0	0	0	0	0	0	0	0	0	0	0	4	33	20	80
IMPG2	0	0	0	0	0	0	0	0	0	0	0	0	0	0	0	0	0	0	0	0	4	28	54	216
KCNV2	0	0	0	0	1	0	2	2	2	4	8	78	0	0	0	0	0	0	0	0	4	22	23	92
LHON	0	0	0	0	0	0	0	0	0	0	0	0	0	0	0	0	0	0	0	0	4	4	22	85
MERTK	2	4	4	196	0	0	0	0	0	0	0	0	0	0	0	0	0	0	0	0	4	30	24	90
MFSD8	0	0	0	0	0	0	0	0	0	0	0	0	0	0	0	0	0	0	0	0	3	31	34	130
MT-TL1	0	0	0	0	0	0	0	0	0	0	0	0	0	0	0	0	0	0	0	0	3	16	17	68

MYO7A	0	0	0	0	1	2	2	2	0	0	0	0	1	2	2	38	2	4	0	20	6	65	39	156
NR2E3	2	4	4	148	2	2	4	4	0	0	0	0	3	6	6	150	0	0	0	0	5	67	42	168
OPA1	1	0	2	74	0	0	0	0	0	0	0	0	0	0	0	0	0	0	0	0	6	12	30	120
PAX6	0	0	0	0	0	0	0	0	0	0	0	0	0	0	0	0	0	0	0	0	3	8	9	36
PCARE	0	0	0	0	0	0	0	0	0	0	0	0	0	0	0	0	0	0	0	0	3	29	22	88
PDE6A	0	0	0	0	1	2	2	2	0	0	0	0	0	0	0	0	0	0	0	0	4	30	39	156
PDE6B	1	2	2	74	0	0	0	0	0	0	0	0	2	4	4	76	0	0	0	0	3	29	35	140
PDE6C	0	0	0	0	0	0	0	0	0	0	0	0	0	0	0	0	0	0	0	0	3	15	47	188
PROM1	14	22	27	760	0	0	0	0	1	0	2	2	2	4	4	94	2	4	0	20	4	35	37	142
PRPF31	2	4	4	244	0	0	0	0	0	0	0	0	3	6	6	114	6	12	0	60	7	23	54	216
PRPF8	0	0	0	0	0	0	0	0	0	0	0	0	0	0	0	0	0	0	0	0	4	25	44	176
PRPH2	41	48	85	3276	5	4	10	10	7	0	14	144	3	6	6	150	23	46	230	230	26	140	175	700
RDH12	2	4	4	160	0	0	0	0	0	0	0	0	0	0	0	0	0	0	0	0	4	17	28	112
RDH5	0	0	0	0	0	0	0	0	0	0	0	0	0	0	0	0	0	0	0	0	3	12	11	44
RHO	10	15	14	524	1	1	2	2	0	0	0	0	10	19	21	416	0	0	0	0	11	64	93	372
RP1	4	6	7	253	4	8	8	8	0	0	0	0	11	22	22	454	7	14	70	70	17	103	134	536
RP1L1	2	4	4	148	2	20	35	705	0	0	0	0	0	0	0	0	0	0	0	0	4	28	22	85
RP2	4	10	7	342	2	0	4	4	0	0	0	0	0	0	0	0	0	0	0	0	5	84	23	92
RP9	0	0	0	0	0	0	0	0	0	0	0	0	0	0	0	0	0	0	0	0	6	61	65	257
RPE65	1	2	2	74	1	2	2	2	0	0	0	0	0	0	0	0	0	0	0	0	4	15	30	120
RPGR	9	18	18	882	6	19	18	18	0	0	0	0	7	14	14	278	0	0	0	0	26	194	394	1567
RS1	6	6	12	556	1	0	2	2	0	0	0	0	7	14	14	290	6	12	60	60	15	186	162	645
SNRNP200	0	0	0	0	0	0	0	0	0	0	0	0	0	0	0	0	0	0	0	0	3	14	17	68
TIMP3	0	0	0	0	0	0	0	0	0	0	0	0	2	4	4	100	0	0	0	0	4	45	41	164
TULP1	0	0	0	0	1	4	2	2	0	0	0	0	0	0	0	0	0	0	0	0	5	40	48	192
TYR	0	0	0	0	0	0	0	0	0	0	0	0	0	0	0	0	0	0	0	0	3	7	2	8
USH1C	2	3	5	185	0	0	0	0	0	0	0	0	0	0	0	0	0	0	0	0	3	9	26	104
USH2A	20	28	28	916	4	16	8	8	0	0	0	0	40	39	41	856	20	40	200	200	46	290	458	1832
WFS1	0	0	0	0	0	0	0	0	0	0	0	0	0	0	0	0	0	0	0	0	3	0	17	68
**Total**	**390**	**633**	**756**	**27756**	**60**	**166**	**204**	**1123**	**40**	**111**	**141**	**1242**	**156**	**268**	**272**	**5634**	**129**	**258**	**1290**	**1290**	**524**	**3986**	**4862**	**19326**

Italic is used for gene names.

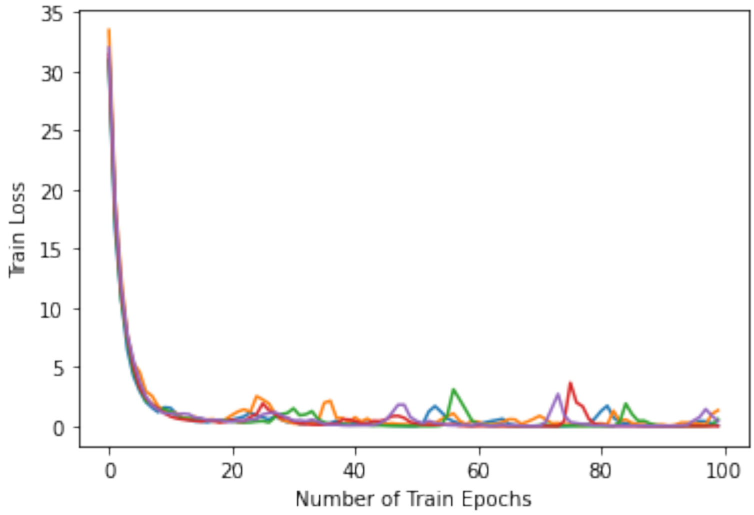

Supplementary Figure 18: Example network training loss curves. 100 epochs was found to be sufficient for training to converge for a wide variety of hyper-parameter settings in preliminary investigations.

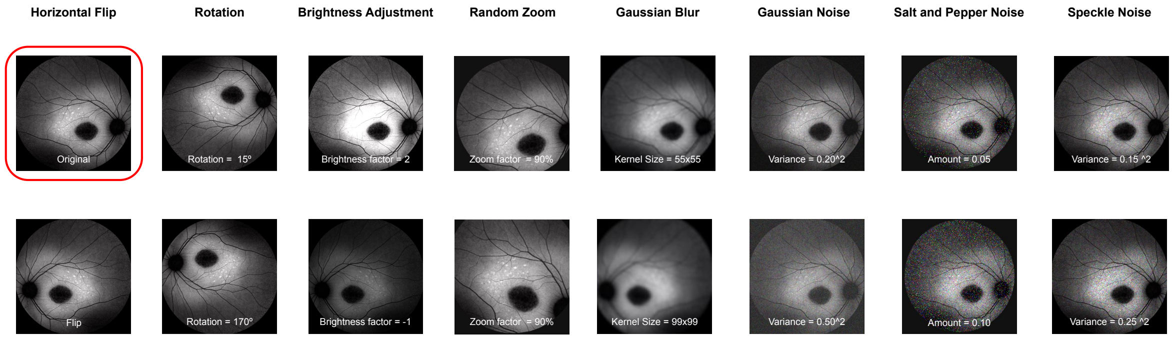

Supplementary Figure 19: Data augmentation transformations applied to the training set. Data augmentation techniques were applied to the training data which were felt to be realistic transformations of the data. These included random brightness adjustment, horizontal flip for all three modalities and rotations by up to 15 degrees.

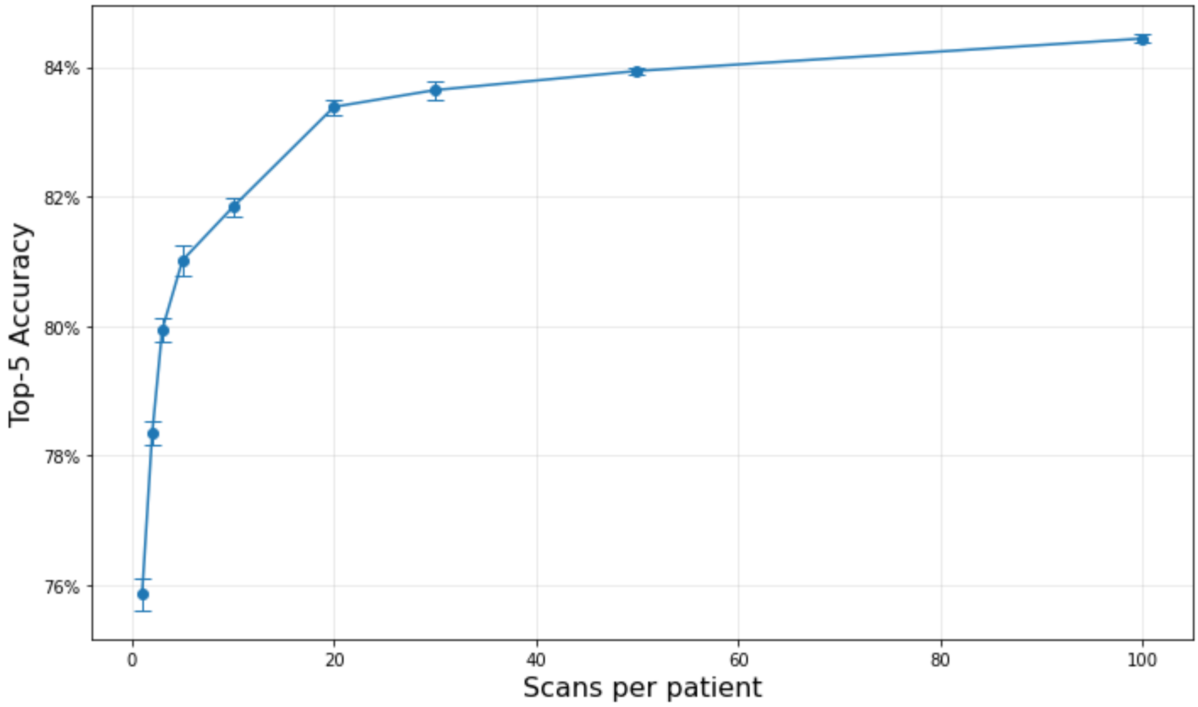

Supplementary Figure 20: Top-5 accuracy of Eye2Gene on the all test data subsamling k images per patient was randomly subsampled (mean over 10 trials) for different values of k (k=1,2,3,5,10,20,30,50,100).

Supplementary Table 7: Additional metrics on full validation data.

**Metrics**		
Top-k	1	64.5%
	2	73.2%
	3	78.8%
	5	83.9%
	10	89.2%
Mean F1		0.478
Weighted F1		0.595
Mean MAP		0.545
Mean AUROC		0.914

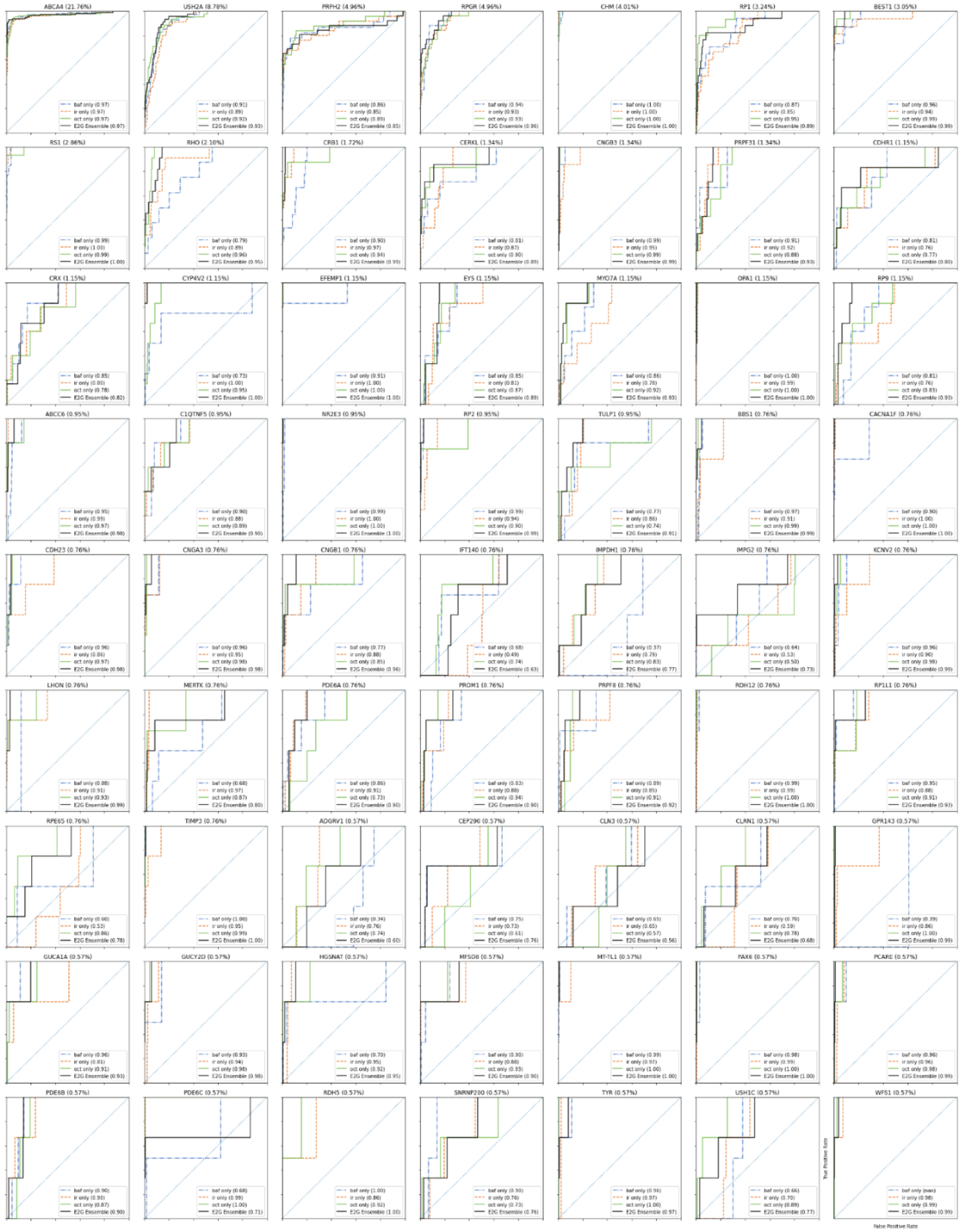

Supplementary Figure 21: Per-gene Receiver Operating Characteristic (ROC) curves of Eye2Gene on the Moorfields Eye Hospital internal test data for the three different imaging modalities across the 63 different predicted genes. Percentages next to gene name denote the percentage of total patients corresponding to the given gene.

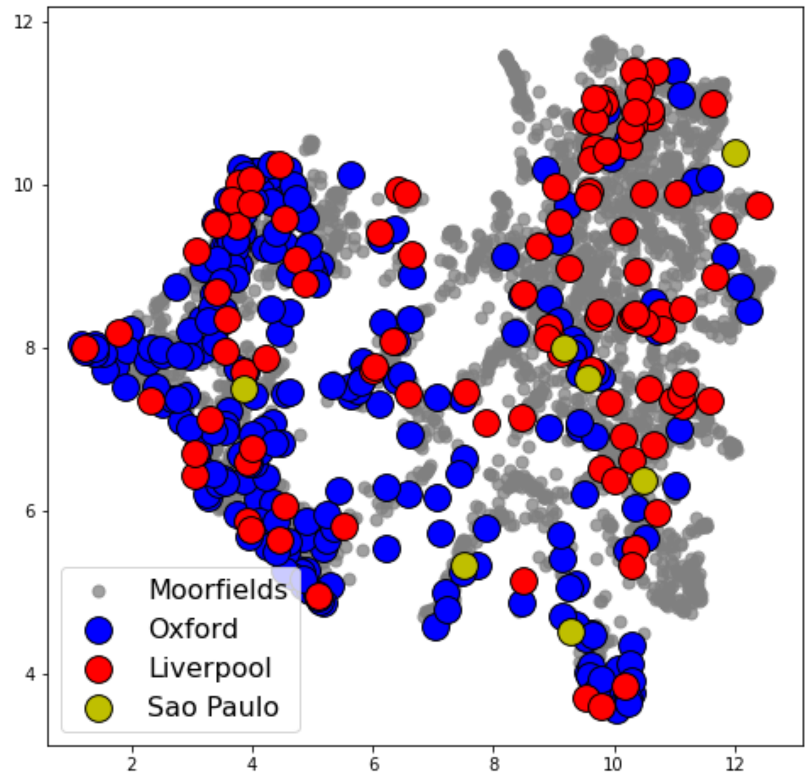

Supplementary Figure 22: UMAP-projected embeddings of FAF images from external sites overlaid over the internal MEH embeddings. Points were projected using the UMAP model derived from the Moorfields data using the python umap-learn package.

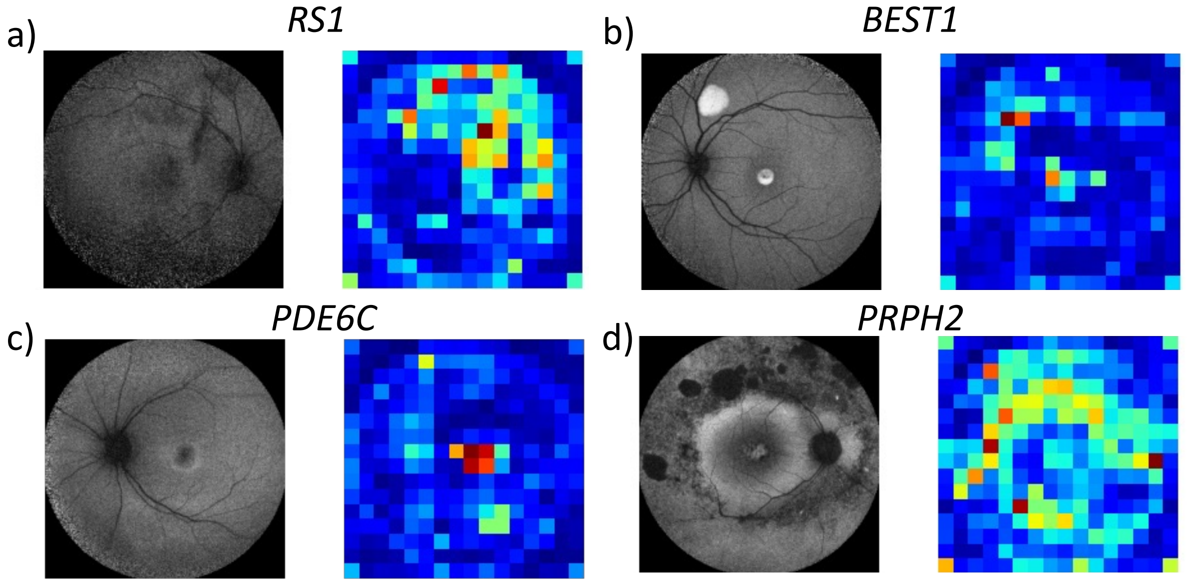

Supplementary Figure 23: Autofluorescence images and corresponding attention heatmaps produced by Eye2Gene (a) *RS1* retinopathy is typically characterized by macular schisis which appears as reduced autofluorescence due to structural disruption of the retinal layers and is highlighted in the heatmap by the orange and red pixels. **(b)** *BEST1* retinopathy is characterized by vitelliform areas of increased autofluorescence and are highlighted on the heatmap by the orange and red pixels **(c)** *PDE6C* retinopathy characterized by reduced autofluorescence at the fovea and a bright ring of autofluorescence due to severe cone dysfunction/loss, which are highlighted by the red and orange pixels on the corresponding heatmap. **(d)** *PRPH2* retinopathy is characterized by dispersed peripheral focal areas of hypo and a ring-like hyper-autofluorescence which are highlighted by the red and orange pixels in the corresponding heatmap.

References

1. Fujinami-Yokokawa, Y. *et al.* Prediction of Causative Genes in Inherited Retinal Disorders from Spectral-Domain Optical Coherence Tomography Utilizing Deep Learning Techniques. *J. Ophthalmol.* **2019**, 1691064 (2019).
2. Shah, M., Roomans Ledo, A. & Rittscher, J. Automated classification of normal and Stargardt disease optical coherence tomography images using deep learning. *Acta Ophthalmol.* **98**, e715–e721 (2020).
3. Miere, A. *et al.* Deep Learning-Based Classification of Inherited Retinal Diseases Using Fundus Autofluorescence. *J. Clin. Med. Res.* **9**, (2020).
4. Fujinami-Yokokawa, Y. *et al.* Prediction of causative genes in inherited retinal disorder from fundus photography and autofluorescence imaging using deep learning techniques. *Br. J. Ophthalmol.* (2021) doi:10.1136/bjophthalmol-2020-318544.
5. Miere, A. *et al.* Deep Learning to Distinguish ABCA4-Related Stargardt Disease from PRPH2-Related Pseudo-Stargardt Pattern Dystrophy. *J. Clin. Med. Res.* **10**, (2021).
6. Chen, C. *et al.* This looks like that: Deep learning for interpretable image recognition. *arXiv [cs.LG]* (2018).
7. Hanany, M., Rivolta, C. & Sharon, D. Worldwide carrier frequency and genetic prevalence of autosomal recessive inherited retinal diseases. *Proc. Natl. Acad. Sci. U. S. A.* **117**, 2710–2716 (2020).
8. Yang, L. *et al.* Genetic Spectrum of EYS-associated Retinal Disease in a Large Japanese Cohort: Identification of Disease-associated Variants with Relatively High Allele Frequency. *Sci. Rep.* **10**, 5497 (2020).
9. Naik, G. *et al.* Retinograd-AI: An open-source automated Fundus Autofluorescence retinal image gradability assessment for Inherited Retinal Dystrophies. *bioRxiv* 2024.08.07.24311607 (2024) doi:10.1101/2024.08.07.24311607.
10. Georgiou, M. *et al.* Prospective Cohort Study of Childhood-Onset Stargardt Disease: Fundus Autofluorescence Imaging, Progression, Comparison with Adult-Onset Disease, and Disease Symmetry. *Am. J. Ophthalmol.* **211**, 159–175 (2020).
11. Glinton, S. L. *et al.* Phenotyping of ABCA4 Retinopathy by Machine Learning Analysis of Full-Field Electroretinography. *Transl. Vis. Sci. Technol.* **11**, 34 (2022).
12. Casalino, G. *et al.* Autosomal Recessive Bestrophinopathy: Clinical Features, Natural History, and Genetic Findings in Preparation for Clinical Trials. *Ophthalmology* **128**, 706–718 (2021).
13. Georgiou, M. *et al.* Adaptive Optics Retinal Imaging in CNGA3-Associated Achromatopsia: Retinal Characterization, Interocular Symmetry, and Intrafamilial Variability. *Invest. Ophthalmol. Vis. Sci.* **60**, 383–396 (2019).
14. Varela, D. CRB1-Associated Retinal Dystrophies: Genetics, Clinical Characteristics, and Natural History. *Am. J. Ophthalmol* **246**, 107–121 (2022).
15. Bouzia, Z. *et al.* GUCY2D-Associated Leber Congenital Amaurosis: A Retrospective Natural History Study in Preparation for Trials of Novel Therapies. *Am. J. Ophthalmol.* (2019) doi:10.1016/j.ajo.2019.10.019.
16. Hahn, L. C. The Natural History of Leber Congenital Amaurosis and Cone-Rod Dystrophy Associated with Variants in the GUCY2D Gene. *Ophthalmol Retina* **6**, 711–722 (2022).
17. Georgiou, M. KCNV2-associated Retinopathy: Genetics, Electrophysiology and Clinical Course - KCNV2 Study Group Report 1. *Am. J. Ophthalmol* (2020).
18. Ba-Abbad, R. *et al.* Macula-predominant retinopathy associated with biallelic variants in RDH12. *Ophthalmic Genet.* **41**, 612–615 (2020).
19. Muthiah, M. N. *et al.* Novel disease-causing variant in RDH12 presenting with autosomal dominant retinitis pigmentosa. *Br. J. Ophthalmol.* **106**, 1274–1281 (2022).
20. Georgiou, M. *et al.* RP2-associated X-linked Retinopathy: Clinical Findings, Molecular Genetics, and Natural History. *Ophthalmology* (2022) doi:10.1016/j.ophtha.2022.11.015.
21. Kumaran, N. *et al.* Retinal Structure in RPE65-Associated Retinal Dystrophy. *Invest. Ophthalmol. Vis. Sci.* **61**, 47 (2020).

22. Anikina, E. *et al.* Characterization of Retinal Function Using Microperimetry-Derived Metrics in Both Adults and Children With RPGR-Associated Retinopathy. *Am. J. Ophthalmol.* **234**, 81–90 (2022).
23. Tee, J. J. L. *et al.* Natural History Study of Retinal Structure, Progression, and Symmetry Using Ellipzoid Zone Metrics in RPGR-Associated Retinopathy. *Am. J. Ophthalmol.* **198**, 111–123 (2019).
24. Georgiou, M. *et al.* X-Linked Retinoschisis: Deep Phenotyping and Genetic Characterization. *Ophthalmology* **129**, 542–551 (2022).
