# Supplementary material for: Next-generation phenotyping of inherited retinal diseases from multimodal imaging with Eye2Gene
Source: Nat Mach Intell. 2025 Jun 18;7(6):967–78. doi: 10.1038/s42256-025-01040-8 (PMC12185311; doi:10.1038/s42256-025-01040-8)
Supplement: Supplementary file 2 — Reporting Summary [file 42256_2025_1040_MOESM2_ESM.pdf]

nature portfolio

Corresponding author(s): Nikolas Pontikos

Last updated by author(s): Sep 26, 2024

Reporting Summary

Nature Portfolio wishes to improve the reproducibility of the work that we publish. This form provides structure for consistency and transparency in reporting. For further information on Nature Portfolio policies, see our Editorial Policies and the Editorial Policy Checklist.

Statistics

For all statistical analyses, confirm that the following items are present in the figure legend, table legend, main text, or Methods section.

n/a	Confirmed	
☐	☒	The exact sample size (*n*) for each experimental group/condition, given as a discrete number and unit of measurement
☐	☒	A statement on whether measurements were taken from distinct samples or whether the same sample was measured repeatedly
☐	☒	The statistical test(s) used AND whether they are one- or two-sided<br>*Only common tests should be described solely by name; describe more complex techniques in the Methods section.*
☐	☒	A description of all covariates tested
☐	☒	A description of any assumptions or corrections, such as tests of normality and adjustment for multiple comparisons
☐	☒	A full description of the statistical parameters including central tendency (e.g. means) or other basic estimates (e.g. regression coefficient) AND variation (e.g. standard deviation) or associated estimates of uncertainty (e.g. confidence intervals)
☐	☒	For null hypothesis testing, the test statistic (e.g. *F*, *t*, *r*) with confidence intervals, effect sizes, degrees of freedom and *P* value noted<br>*Give P values as exact values whenever suitable.*
☒	☐	For Bayesian analysis, information on the choice of priors and Markov chain Monte Carlo settings
☐	☒	For hierarchical and complex designs, identification of the appropriate level for tests and full reporting of outcomes
☐	☒	Estimates of effect sizes (e.g. Cohen's *d*, Pearson's *r*), indicating how they were calculated

Our web collection on statistics for biologists contains articles on many of the points above.

Software and code

Policy information about availability of computer code

Data collection	Retrospective deidentified imaging data and population characteristics were collected via the VisionSense eCRF (https://grading.readingcentre.org) as per the study protocol: https://liveuclac-my.sharepoint.com/:w:/g/personal/rmhanpo_ucl_ac_uk/EZmNh0tHYHtBgWfHr-Tj758BW0R7sb1t7HvOI19kwT8T4A?e=EhzFaE<br>All the software relating to this project is under the Github organisation: https://github.com/Eye2Gene/.<br>The code for training/testing the classification model is in the private Github repository: https://github.com/Eye2Gene/Classification.<br>The running version of the web app is accessible at https://app.eye2gene.com and users are able to login with the following credentials:<br>user: reviewer@nature.com<br>password: naturemedicine1995
Data analysis	The Eye2Gene model was trained and evaluated in Python 3.10.2 (www.python.org) and Keras/Tensorflow 2.15.0 (https://keras.io/) with the keras-cv-attention-models v1.3.19 library from PyPi (https://github.com/0723sjp/keras_cv_attention_models). All training and prediction code is available at https://github.com/Eye2Gene/Classification. The code can also be ran online via the CodeOcean capsule (https://codeocean.com/capsule/0706698/).<br>Results were analysed in JupyterLab 3.2.8 (https://jupyter.org/) with Python 3.9.7 (https://www.python.org/), NumPy 1.25.0 (https://numpy.org/), Pandas 1.4.0 (https://pandas.pydata.org/), SciPy 1.12.0 (https://scipy.org/), Scikit-Learn 1.0.2 (https://scikit-learn.org/), and MatplotLib 3.5.1 (https://matplotlib.org/).

For manuscripts utilizing custom algorithms or software that are central to the research but not yet described in published literature, software must be made available to editors and reviewers. We strongly encourage code deposition in a community repository (e.g. GitHub). See the Nature Portfolio guidelines for submitting code & software for further information.

Data

Policy information about availability of data

All manuscripts must include a data availability statement. This statement should provide the following information, where applicable:

- Accession codes, unique identifiers, or web links for publicly available datasets
- A description of any restrictions on data availability
- For clinical datasets or third party data, please ensure that the statement adheres to our policy

The data that support the findings of this study are divided into two groups, published data and restricted data. Published data constitutes synthetic data derived from the Eye2Gene training dataset and are available from Figshare at https://doi.org/10.5522/04/28604234.v1 50. In combination with the code at https://github.com/Eye2Gene/Classification 49, this can be used to train a smaller local version of Eye2Gene. Restricted data are curated for Eye2Gene users under a license and cannot be published, to protect patient privacy and intellectual property. Access request to Eye2Gene datasets for the purpose of collaboration can be made via a contact form on the Eye2Gene website (www.eye2gene.com).

Human research participants

Policy information about studies involving human research participants and Sex and Gender in Research.

Reporting on sex and gender	Gender information has been collected for the participants as recorded in their clinical record.
Population characteristics	Age at presentation, self-reported ethnicity, gender, mode of inheritance of condition and diagnostic gene associated with the condition.
Recruitment	Retrospective deidentified data collected during standard clinical care.
Ethics oversight	This research was approved by the IRB and the UK Health Research Authority Research (HRA) Ethics Committee (REC) reference (22/WA/0049) "Eye2Gene: accelerating the diagnosis of inherited retinal diseases" Integrated Research Application System (IRAS) (project ID: 242050). The study sponsor was the University College London Joint Research Office (UCL JRO). The UCL JRO Data Protection reference number is Z6364106/2021/11/67. A summary of the research study can be found on the HRA website (https://www.hra.nhs.uk/planning-and-improving-research/application-summaries/research-summaries/eye2gene-10/). The REC that approved this study is Wales REC 5 (Wales.REC5@Wales.nhs.uk). All research adhered to the tenets of the Declaration of Helsinki.

Note that full information on the approval of the study protocol must also be provided in the manuscript.

Field-specific reporting

Please select the one below that is the best fit for your research. If you are not sure, read the appropriate sections before making your selection.

☒ Life sciences ☐ Behavioural & social sciences ☐ Ecological, evolutionary & environmental sciences

For a reference copy of the document with all sections, see nature.com/documents/nr-reporting-summary-flat.pdf

Life sciences study design

All studies must disclose on these points even when the disclosure is negative.

Sample size	Since we are dealing with a rare condition, the entirety of the IRD data available at Moorfields Eye Hospital was use for the training.
Data exclusions	Participants were excluded based on availability/quality of imaging or missing genetic diagnosis information. The participant list was further filtered to only include participants with a gene diagnosis in one of 63 genes for training the AI model.
Replication	External validation was conducted in 4 external datasets.
Randomization	During the Eye2Gene AI training process participants were randomized to 5 different folds.
Blinding	Human benchmarking and evaluation of the Eye2Gene AI algorithm involved blinding the expert ophthalmologists and the AI algorithm to the known diagnostic gene when presented with a retinal scan.

Reporting for specific materials, systems and methods

We require information from authors about some types of materials, experimental systems and methods used in many studies. Here, indicate whether each material, system or method listed is relevant to your study. If you are not sure if a list item applies to your research, read the appropriate section before selecting a response.

Materials & experimental systems

n/a	Involved in the study
☒	☐ Antibodies
☒	☐ Eukaryotic cell lines
☒	☐ Palaeontology and archaeology
☒	☐ Animals and other organisms
☐	☒ Clinical data
☒	☐ Dual use research of concern

Methods

n/a	Involved in the study
☒	☐ ChIP-seq
☒	☐ Flow cytometry
☒	☐ MRI-based neuroimaging

Clinical data

Policy information about clinical studies

All manuscripts should comply with the ICMJE guidelines for publication of clinical research and a completed CONSORT checklist must be included with all submissions.

Clinical trial registration	Z6364106/2021/11/67, UCL Data Protection reference number
Study protocol	The study protocol is available for download here: https://liveuclac-my.sharepoint.com/:w:/g/personal/rmhanpo_ucl_ac_uk/EZmNh0tHYHtBgWfHr-Tj758BW0R7sb1t7HvOI19kwT8T4A?e=EhzFaE
Data collection	Retrospective de-identified data collection over 2006-06-05 to 2018-04-05.
Outcomes	The measured outcomes are the Eye2Gene AI model prediction top-5 accuracy for identifying the correct diagnostic gene based on retinal scans. We also compare the ranking of the correct gene between two methods.
